# Homologous recombination deficiency in primary ER-positive and HER2-negative breast cancer

**DOI:** 10.1038/s43856-026-01385-0

**Published:** 2026-02-16

**Authors:** Helen R. Davies, Daniella Black, Anders Kvist, Kristín Sigurjónsdóttir, Ana Bosch, Ramsay Bowden, Yasin Memari, Ziqian Chen, Giuseppe Rinaldi, Frida Rosengren, Deborah F. Nacer, Srinivas Veerla, Lennart Hohmann, Nicklas Nordborg, Jari Häkkinen, Johan Vallon-Christersson, Åke Borg, Serena Nik-Zainal, Johan Staaf

**Affiliations:** 1 Department of Genomic Medicine, School of Clinical Medicine & Early Cancer Institute, University of Cambridge, Cambridge, UK; 2 Division of Oncology, Department of Clinical Sciences Lund, Lund University, Medicon Village Lund, Sweden; 3 Division of Translational Cancer Research, Department of Laboratory Medicine, Lund University, Medicon Village Lund, Sweden; 4 Department of Oncology, Skåne University Hospital, Lund, Sweden

**Keywords:** Breast cancer, Prognostic markers, Cancer genetics, Cancer genomics, Cancer epigenetics

## Abstract

**Background:**

Homologous recombination deficiency (HRD) originating from inactivation of genes like *BRCA1*/*BRCA2* is a targetable abnormality common in triple-negative breast cancer (TNBC). In estrogen-receptor (ER)-positive HER2-negative (ERpHER2n) breast cancer (BC), HRD prevalence and clinical impact are unclear.

**Methods:**

We analyzed 502 ERpHER2n tumors from patients recruited via the population-representative Swedish SCAN-B study by whole genome sequencing (WGS), defining mutational signatures-based HRD, as well as matched transcriptional, DNA methylation, clinicopathological, adjuvant treatment, and outcome data.

**Results:**

We show that HRD is much less frequent in ERpHER2n BC (8.4%) compared to TNBC, though induced by similar genetic/epigenetic mechanisms acting on mainly *BRCA1*/*BRCA2*/*RAD51C*/*PALB2* together, providing a plausible HR-inactivation mechanism for 71.4% of HRD tumors. Our modelled estimate of HRD in Western European/Nordic BC is ~10-13%. HRD tumors were observed across all PAM50 gene expression subtypes with the exception of Luminal A tumors ( < 1%) and did not exhibit a unique, defining transcriptional or DNA methylation profile. While HRD status was not statistically associated with differences in patient outcome for patients treated with combined chemotherapy and endocrine therapy, a nonsignificant trend of poorer outcome for patients with HRD tumors was observed for patients treated with adjuvant endocrine therapy only.

**Conclusions:**

ERpHER2n HRD tumors show features of aggressive disease, but do not display a distinct transcriptional or DNA methylation profile that clearly differentiates them from HR-proficient tumors. Though numbers are limited, we present early evidence that HRD stratification by WGS could impact therapeutic strategies, as HRD BCs trended to poorer outcomes when not treated with chemotherapy.

## Plain language summary

In this study, we examined the frequency, causes, molecular features, and clinical relevance of homologous recombination (HR) deficiency (HRD) in primary ER-positive/HER2-negative breast cancer using whole-genome sequencing of 502 tumors. HRD was uncommon ( < 10%) and mainly driven by alterations in well-established DNA repair genes, similar to other breast cancer subtypes. HRD tumors did not show clear differences in gene expression or DNA methylation compared with HR-proficient tumors. Clinically, HRD status was not significantly associated with patient outcomes after adjuvant systemic therapy, although patients with HRD tumors treated with endocrine therapy alone showed a trend toward poorer outcomes.

## Abstract (web summary)

Davies, Black et al., investigate the frequency, cause, molecular associations, and prognostic implications of homologous recombination deficiency (HRD) in primary estrogen receptor-positive and HER2-negative breast cancer. HRD was less frequent (8.4%), induced mainly by alterations acting on BRCA1/BRCA2/RAD51C/PALB2, but not associated with patient outcome after adjuvant systemic therapy.

## Introduction

Breast cancers (BC) that are estrogen receptor (ER) positive and human epidermal growth factor receptor 2 (HER2) negative (ERpHER2n) constitute the largest clinical subgroup of primary BC, representing ~70% of all BC cases^1^. The ERpHER2n subgroup is a heterogeneous mix of tumors with varying clinical aggressiveness, treated typically along two main lines: adjuvant endocrine therapy only (Endo) or combined neo/adjuvant systemic chemotherapy and endocrine therapy (ChemoEndo). Current treatment guidelines now suggest complementing ChemoEndo treatment with CDK4/6 inhibitors or potentially PARP inhibitors, depending on *BRCA1/2* germline status for high-risk patients, which has improved invasive disease-free survival at 4 years by 6–7% (see e.g., ref. ^2^). Despite continuous improvements in therapeutic strategies, many patients with primary ERpHER2n BC still relapse. This represents a substantial clinical challenge requiring improved methods of risk assessment and identification of new targets for selective therapeutics. For decades, immunohistochemistry (IHC) and diverse genomic and transcriptomic methods have been explored in ERpHER2n BC, resulting in an array of reported prognostic and/or predictive biomarkers^3,4^. Only a handful have reached clinical implementation and are proven to aid therapeutic decisions in restricted tumor subsets^2,5^.

In parallel to the extensive transcriptional characterization of BC, large-scale whole genome sequencing (WGS) studies have delineated the genomic landscape of BC, pinpointing not only specific oncogenic drivers of this disease but also the broader mutational landscape inferred by mutational processes (e.g., ref. ^6^). One prominent mutational process is homologous recombination (HR) deficiency (HRD), typically caused by disruption of genes involved in the HR pathway, including e.g. *BRCA1*, *BRCA2, PALB2* and *RAD51C*, by various mechanisms including germline and/or somatic mutation and promoter hypermethylation. Tumor HRD classification is typically based on genomic analyses^7^, historically using copy number aberrations or targeted next-generation sequencing (NGS) panels, and more recently through WGS approaches. HRD confers specific patterns of somatic mutations and structural variation (referred to as mutational signatures)^6^ forming the foundation of WGS-based predictors such as HRDetect^8^. Applications of HRD predictors have further unveiled that a substantial proportion of BC displays somatic signatures of HRD, with reports of close to 60% in triple negative breast cancers (TNBCs), some without known pathogenic germline or somatic variants and/or promoter hypermethylation of HR genes^8,9^. Critically, tumors with HRD signatures have been shown to respond favorably to compounds that increase the demand on compensatory repair pathways, such as DNA-damaging agents (e.g., platinum) and PARP inhibitors, irrespective of HR gene status (e.g., refs. ^10–14^), with the strongest clinical trial data support mainly in ovarian cancer^15,16^.

In primary BC, current guidelines recommend adjuvant PARP-inhibitor therapy for patients with germline pathogenic variants in *BRCA1/BRCA2*, but not yet for tumors with an HRD phenotype per se^2^. In part, this may be due to conflicting results about the association of HRD with treatment response caused by the variation in how HRD is defined and measured (see refs. ^17,18^). A genomic HRD classification may not necessarily correlate to active HRD in a tumor, as reports have shown that prolonged treatment with platinum-based or PARP-inhibitor therapies can result in the selection of resistance-causing reversion mutations or gene reactivation by promoter demethylation restoring HR^19,20^. However, functional HRD assays, like the RAD51 assay, also have multiple practical limitations for clinical use (see ref. ^7^), and the degree of concordance of HRD status between genetic classifiers and functional HRD assays in primary treatment-naïve patients, while promising, is not fully established^11^.

Unlike in TNBC, the landscape of HRD in the most common BC subtype of ERpHER2n remains unclear. In the earliest days of NGS, tumors that were sequenced were not representative of a more general patient population^8,21–23^. Moreover, systematic clinical data collection was lacking, and thus the relationship between HRD status, treatment, and patient outcome, especially on standard-of-care (SOC) therapy, has not been robustly interrogated. Finally, varying frequencies of HRD-positivity in BC have been reported^8,22,24^, dependent both on differences in HRD-calling approach and cohort compositions.

In this study, we investigate the largest to date reported cohort of WGS analyzed ERpHER2n BC (*n* = 502) within a SOC therapy setting involving adjuvant chemotherapy and endocrine therapy by taking advantage of the prospective, population-representative, Sweden Cancerome Analysis Network – Breast (SCAN-B) study^25,26^ providing long-term clinical follow-up. In addition to WGS, RNA-sequencing, global DNA methylation, and complete clinical follow-up data are also included. Here, we investigate the frequency, causes, molecular associations, and prognostic implications of HRD in primary ERpHER2n BC collected in a routine diagnostic setting.

## Methods

### Inclusion and Ethics statement

Patients with primary ERpHER2n BC were enrolled in the SCAN-B study (NCT02306096) during 2010-2014. Ethical approval was given for the SCAN-B study (Registration numbers 2009/658, 2010/383, 2012/58, 2013/459, 2014/521, 2015/277, 2016/541, 2016/742, 2016/944, 2018/267, 2019/01252, and 2024-02040-02) by the Regional Ethical Review Board in Lund, Sweden, governed by the Swedish Ethical Review Authority, Box 2110, 750 02 Uppsala, Sweden. All patients provided written informed consent prior to enrolment, including to publish information about sex and age. All analyses were performed in accordance with patient consent and ethical regulations and decisions. Patient gender was not considered as an inclusion or exclusion criteria for the study. This study conformed to the principles of the Helsinki Declaration.

### SCAN-B ERpHER2n breast cancer cohort

Between September 2010 and the end of 2014, 3066 patients were diagnosed with primary ERpHER2n breast cancer in the Skåne Healthcare region, Sweden, based on data from the Swedish national breast cancer registry. Of these, 2611 were enrolled in the SCAN-B study, and 1834 of the 2611 (70.2%) patients were included in the study by Staaf et al.^27^, based on the availability of RNA-sequencing data (including PAM50 subtype classification). Further inclusion and exclusion criteria for the 1834 patients are described in detail in Staaf et al.^27^, including clinical data provided from the national breast cancer registry. The ERpHER2n patients in the study by Staaf et al. have been shown to be representative of the patient demographics in the total catchment region during the inclusion period^27^. From the 1834 patients, 533 were selected for WGS analysis based on a combination of PAM50 subtype classification, administered treatment based on clinical data, availability, and sufficient amount of tumor and matched normal DNA for WGS. Patients were prioritized for WGS based on: (i) ChemoEndo adjuvant therapy irrespective of PAM50 subtype to allow survival analyses specifically in this treatment group, (ii) a PAM50 Basal, Luminal B (LumB), or HER2-enriched (HER2E) subtype irrespective of adjuvant therapy to allow for comparison of HRD frequency in these PAM50 subtypes, and (iii) an addition of a small set of Luminal A (LumA) tumors. After WGS quality assessment, 31 patients were removed, with the primary cause of WGS failure being  an estimated low tumor cell content in the tumor tissue. The final SCAN-B patient cohort comprised 502 patients with successful WGS (see CONSORT diagram in Supplementary Fig. S1a, Table 1, and Supplementary Data 2 for patient characteristics). Of the 502 patients, 138 received endocrine adjuvant therapy (Endo), forming the final Endo-treated group for survival analyses. Of these 138 patients, all had available overall survival (OS) data, while 136 patients had available distant relapse-free interval (DRFI) data from ref. ^27^. Of the remaining patients, 352 received combined adjuvant chemotherapy and endocrine therapy (ChemoEndo) based on registry data^27^, with 339 of 352 (96.3%) cases confirmed by clinical review to have received chemotherapy, forming the final ChemoEndo group for survival analyses. The main chemotherapeutic strategy was fluorouracil+epirubicin hydrochloride+cyclophosphamide (FEC) combined with a taxane (typically docetaxel) (92.6% of the 339 ChemoEndo patients).

**Table 1:** Clinicopathological characteristics of the ERpHER2n WGS cohort and total HRD frequency

	Total cohort	ChemoEndo^a^	Endo
Number of patients	502 (100%)	339 (67.5%)	138 (27.5%)
Female/male (%)	98.8/1.2%	99.7/0.3%	97.1/2.9%
Median age (years)	65	60	75
Median tumor size and range (mm)	20 (2–115)	20 (2–115)	18 (8–67)
Nottingham Grade (NHG)			
NHG 1	8.1%	6.4%	10.1%
NHG 2	40.0%	35.8%	49.3%
NHG 3	51.8%	57.9%	40.6%
Nodal status			
Node-negative (N0)	47.3%	38.4%	62.3%
Node-positive (N+)	52.7%	61.6%	37.7%
HER2-low status^b^	84.8%	84.6%	85.7%
PR-status (positive/negative)	84.8/15.2%	86.1/13.9%	81.9/18.1%
Adjuvant therapy			
ChemoEndo^a^	71.1%	100%	0%
Endo	28.9%	0%	100%
Complete overall survival data	100%	100%	100%
Complete DRFI survival data^c^	99.6%	98.5%	98.6%
Germline screening data available	11.4%	13.9%	3.6%
HRD frequency	8.4%	9.1%	7.2%
PAM50 subtypes			
Basal	3.3%	2.1%	4.3%
HER2-enriched (HER2E)	6.3%	4.0%	11.6%
Luminal A (LumA)	30.9%	37.1%	18.1%
Luminal B (LumB)	57.7%	54.1%	65.9%
Normal	1.8%	2.7%	0%

Note: Cases with missing values for a variable are excluded from percentage calculations if not otherwise stated.

^a^Only ChemoEndo cases with clinical review data are included.

^b^HER2-low classification was only possible in reviewed cases (including some, but not all, Endo cases). Cases with missing values are omitted.

^c^By clinical review or cancer registry data, depending on the cohort.

In the SCAN-B cohort, ER and progesterone receptor (PR) data are based on routine clinical IHC analysis, while HER2-status was determined from routine clinical IHC and fluorescence in situ hybridization (FISH) analyses as reported in the Swedish national breast cancer quality registry. Specifically, ER-positivity was defined as ≥10% of tumor cells being IHC-stained according to current Swedish national guidelines. HER2-negativity was defined as an IHC HER2-staining score of <2, or for patients with IHC 2+, a non-amplified ISH status. Fresh tumor tissue from SCAN-B patients was preserved in RNAlater™ Stabilization Solution (Invitrogen) at −80 °C as outlined^25^. Briefly, all tumor tissue used for this study was collected at the time of surgery prior to any treatment start, i.e., representing treatment-naïve specimens. RNA and DNA were isolated from each tissue piece as described^25–27^, and used for RNA-sequencing (RNA), WGS (DNA), and DNA methylation analysis (DNA). No patients were tested by commercial, clinically approved, gene expression-based assays in a routine diagnostic setting, as these were not included in national treatment guidelines during the inclusion years. Records of germline screening were obtained from the study by Nacer et al.^28^. Patients had been referred to counseling and genetic screening according to the practitioner’s choice based on at-the-time current guidelines. The individual cause for a patient’s referral to screening was not accessible due to ethical permissions, and only the clinically reported result was available for the study. Patients were analyzed for germline pathogenic variants in selected genes mainly through NGS-based hybrid capture panels^29^ that include 11 genes previously associated with breast cancer^30,31^: *ATM*, *BARD1*, *BRCA1*, *BRCA2*, *CDH1*, *CHEK2*, *PALB2*, *PTEN*, *RAD51C*, *RAD51D*, and *TP53*. Explicit details are provided in Nacer et al.^28^.

### Non-related ERpHER2n gene expression SCAN-B cohort

From the study by Staaf et al.^27^, 4924 ERpHER2n tumors diagnosed 2010-2018, including all but five WGS cases from the 502-sample set due to lack of lymph node registry data, were available, providing proportions of PAM50 subtypes in ERpHER2n disease. Of these, 4427 did not overlap with the WGS cohort and were defined as eligible for follow-up, with clinical data, including lymph node status, obtained from ref. ^27^. From the set of 4427 patients, PAM50 subtype classification, clinical data, and DRFI data were available for 2046 Endo-only treated patients and 761 ChemoEndo treated patients. For these patients, HRD status is unknown, while RNA-sequencing and PAM50 subtyping were performed identically to the WGS analyzed cohort. Clinical and outcome data for these patients are based on cancer registry information from^27^.

### HRD estimate in HER2-positive BC

In the study by Nik-Zainal et al.^6^, 73 HER2-amplified tumors were analyzed by WGS, with three cases being called HRD (4.1%) by HRDetect^8^.

### Gene expression analyses

RNA-sequencing data were available as fragments per kilobase million (FPKM) values from^27^ for all patients, including also PAM50 molecular subtypes (referred to as NCN subtypes in ref. ^27^) and Risk Of Recurrence (ROR) risk groups (referred to as NCN-ROR-T0 in ref. ^27^) both based on nearest centroid classifications. Based on FPKM data, gene expression-based rank scores for eight biological metagenes in breast cancer originally defined by Fredlund et al.^32^ (termed basal, lipid, mitotic progression, mitotic checkpoint, immune response, early response, steroid response, and stroma) were calculated as described by Nacer et al.^28^. Rank scores were computed individually for each tumor from FPKM data without any further normalization or data centering.

Differential, supervised, gene expression analysis was performed using Student t-test on FPKM data that were (i) offset by +0.1 and (ii) log2-transformed, and False Discovery Rate (FDR)-adjusted for multiple testing using the *p.adjust()* R function. Significant genes were further required to have a mean FPKM > 1 in both test groups to exclude genes with statistical significance but very low expression overall. Principal component analysis (PCA) was performed using the R function *prcomp()* with the parameters center = TRUE, scale = TRUE, retx = TRUE. In the analysis, FPKM data were offset by +0.1 and log2-transformed prior to the PCA.

Matched CIBERSORTx immune cell type deconvolution based on RNA-sequencing data was obtained from the study by Nacer et al.^28^, using a *p*-value cut-off of *p* < 0.05 as described. T-cell receptor (TCR) and B-cell receptor (BCR) repertoire analyses were performed using RNA-sequencing fastq files for SCAN-B tumors processed with *MiXCR* (v4.5.0) using the *rna-seq* preset and specifying species *hsa*^33,34^. For the divergence analysis, we used *post-analysis* and *individual* presets, enabling the calculation of CDR3 metrics, Shannon-Wiener diversity measurement, and gene segment usage for all tumors as a single group.

Pathway analysis was performed using the R ClusterProfiler package (v4.12.6)^35^ and the R package implementations of KEGG, Gene Ontology (org.Hs.eg.db, v3.19.1), Reactome (ReactomePA v1.48.0), and Molecular Signatures Database (MsigDB, msigdbr v10.0.1) gene sets associated with Gene Set Enrichment Analysis (GSEA), as outlined in the ClusterProfiler vignette for the respective analysis. An adjusted *p*-value < 0.05 was used as the significance threshold in all analyses. A list including only significant genes was used as input to the analyses. If a gene universe was required, then the full set of 19,675 genes for which FPKM data were available was used.

Gene expression-based HRD classification was performed using the 228-gene HRD nearest centroid classifier (comprising one HRD centroid and one HR-proficient centroid, referred to as TS228) reported by Jacobson et al.^36^. Prior to classification using FPKM data, an offset of 0.1 was added, the data were log2-transformed, and mean-centered. For each sample, the Pearson correlation to each centroid and an HRD score were computed as outlined by Jacobson et al.^36^. Classification was performed using either the highest correlation or specific correlation cut-offs applied to the HRD centroid values.

### Whole genome sequencing analyses and HRD prediction

For each SCAN-B patient, a tumor sample taken at surgery or as a core biopsy and matched blood DNA were sequenced at Novogene (Novogene, UK) using 150 bp paired end sequencing on an Illumina Novaseq to reach 120-150 GB of sequence, resulting in a tumor coverage between 26-50x (median 36x) after duplicate removal and final filtering. Resulting BAM files were aligned to the reference human genome (GRCh38) using dockstore-cgpmap (v3.2.0), implementing bwa mem (v0.7.17-r1188). Mutation calling of WGS data for SCAN-B tumors was performed as described previously^6^. Briefly, the mutation calling pipeline was containerized within dockstore-cgpwgs (v2.1.1, https://quay.io/repository/wtsicgp/dockstore-cgpwgs), implementing Caveman (Cancer Variants through Expectation Maximization, v1.13.15, http://cancerit.github.io/CaVEMan/) for somatic substitution calling, Pindel (v3.2.0, http://cancerit.github.io/cgpPindel/) for detection of somatic small insertions and deletions (indels), and BRASS (BReakpoint AnalySiS, https://github.com/cancerit/BRASS) for structural rearrangements. ASCAT (v4.2.1, https://github.com/cancerit/ascatNgs) was used to supply average ploidy and purity inputs for Caveman. Additional filtering was applied as follows: single base substitutions (SBSs) were filtered based on a PASS criterion, CLPM = 0.00, and ASMD > = 140, small indels were filtered by QUAL > = 250 & REP < 10, and structural variants filtered for BRASS assembly score >0 indicating successful de novo local assembly using Velvet to determine exact coordinates and features of breakpoint junction sequence.

Mutational signatures were assigned using Fit Multi-Step (FitMS, https://github.com/Nik-Zainal-Group/signature.tools.lib)^37^. For substitution signatures, reference SBSs previously identified in breast cancer were used in combination with high-confidence rare signatures from any organ. A fixed threshold of 5% of the total mutations contributing to the signature was applied before assigning a signature, and an error reduction of 20% was used. The rare signature assignment was manually reviewed. Structural rearrangement signatures were fitted to all samples with a total number of rearrangements greater than 25 by Fit (https://github.com/Nik-Zainal-Group/signature.tools.lib) using reference signatures previously identified in breast cancer. In addition, signatures were only assigned if a minimum of 5 variants and a minimum percentage of 5% of the total rearrangements were contributing to the signature. The resulting signatures were used as input to HRDetect^8^.

Somatic mutations were annotated to Ensembl (v91) using VAGrENT (Variation Annotation GENeraTor, https://github.com/cancerit/VAGrENT). Non-synonymous point mutations and small indels were assessed for potential driver mutations by comparison to the list of genes in the Cancer Gene Census (https://cancer.sanger.ac.uk/census) and genes previously identified as breast cancer driver genes from a previous study^6^. Mutations within these genes were considered to be potential drivers if the same mutation exists multiple times in the COSMIC database or if it was reported as pathogenic or likely pathogenic in cancer in ClinVar (www.ncbi.nlm.nih.gov/clinvar/). In addition, mutations in genes that are reported in the Cancer Gene Census as tumor suppressor genes (excluding those genes where the known mechanism of mutation was restricted to gene fusions) were considered to be potential drivers if the mutation was predicted to result in a premature truncation (nonsense, essential splice, frameshift mutations). Loss of all wild-type alleles in tumor suppressor genes due to loss of heterozygosity was assessed using the ASCAT copy number for the corresponding segment.

WGS data were processed for copy number analysis by a modified ASCAT (v3.1.2) approach first described in Hohmann et al.^38^. Necessary reference files for this version are available at https://github.com/VanLoo-lab/ascat/tree/master/ReferenceFiles/WGS. Changes to the ASCAT algorithm are outlined at https://github.com/nnordborg/ascat/tree/scanb (“Changes in the SCANB-fork”). Baseline parameters were imbalance.test=bimodality_coefficient and tau=0.4. ASCAT segments were called as copy number gain or loss, considering the tumor ploidy as described by Staaf et al.^9^.

HRD status was assessed with HRDetect^8^, using ≥0.9 as the probability threshold for calling HRDetect-high, <0.1 as HRDetect-low, and 0.1-0.9 as HRDetect-intermediate, consistent with Black et al.^39^. In analyses involving a two-group comparison, HRDetect-high tumors were considered HR-deficient (HRD or HRD-positive), while HRDetect-low and HRDetect-intermediate tumors were combined into a single HR-proficient group (or HRD-negative). Copy number signature proportions per tumor for 25 signatures (CN1-25) defined by Steele et al.^40^ were calculated using segmented ASCAT data and code available from COSMIC (v3.4, https://cancer.sanger.ac.uk/signatures/cn/). scarHRD HRD scores were calculated based on segmented ASCAT data using R scripts available from the project’s GitHub repository^41^. Tumors with summarized HRD scores >42 were called as HRD, otherwise HR-proficient. Classifier of HOmologous Recombination Deficiency (CHORD)^24^ HRD classification was performed by first combining the Caveman (single nucleotide variants, SNVs) and Pindel (indel) VCF files into one common SNV VCF file per sample with Bcftools “concat” with option “-a”^42^. After the merge, only variants tagged PASS were retained. The BRASS VCF files containing structural variants (SVs) were used as is. HR deficiency was predicted with CHORD in single-sample mode using the merged SNV and BRASS SV files as input for each sample. The reference genome was GRCh38 and the option “-include_non_pass” was used to retain all variants in the BRASS VCF in CHORD prediction (BRASS does not tag variants as PASS/noPASS, and the PASS tag is required by CHORD). Human Leukocyte Antigen (HLA) analysis was performed using ASCAT data and the HLA*LA-HLA typing software^43^. Detected alleles were filtered using the variables perfectG = =1 and proportionkMersCovered = =1.

### DNA methylation analyses

Matched Illumina MethylationEPIC v1.0 DNA methylation profiles for 499 of 502 tumors were obtained from Gene Expression Omnibus accession GSE278586^38^. Methylation profiling of the SCAN-B cohort was performed by the SNP&SEQ Technology Platform in Uppsala (www.genotyping.se). The facility is part of the National Genomics Infrastructure supported by the Swedish Research Council for Infrastructures and Science for Life Laboratory, Sweden. Beta values, representing the level of methylation, were computed in a sample-by-sample context using the minfi R package (v1.44) function *preprocessNoob()*, Infinium probe normalized using the approach described in ref. ^44^ and filtered according to Aine et al.^45^, leaving 741144 CpGs for analyses. Promoter methylation status of HR-associated genes was assessed based on promoter-associated CpGs using manual inspection of normalized beta values.

For unsupervised and supervised analyses we used beta values adjusted for tumor purity as conceptually outlined by ref. ^46^. Adjustment of beta values for tumor purity using the PureBeta pipeline^47^ (referred to as tumor purity-adjusted beta values) was performed using WGS-based tumor purity estimates (ASCAT values) as input and the previously normalized beta values. We applied the function with default parameters (including the refitting option set to false) and used reference CpG models previously established by Aine et al.^45^ in TNBC. Unsupervised PCA was performed using the R function *prcomp()* with the parameters center = TRUE, scale = TRUE, retx = TRUE on tumor purity-adjusted beta values. For identifying differentially methylated CpGs, tumor purity-adjusted beta values were used as a starting point. We first searched for differentially methylated CpGs comparing beta values using the Wilcoxon test for two-group comparisons or the Kruskal-Wallis test if more than two test groups. For each CpG, if the standard deviation in a comparison was 0, then the *p*-value was set to 1. When filtering CpGs, we established a minimum absolute difference between mean beta per test group (>0.25) as an additional requirement to avoid obtaining significantly differentially methylated CpGs with very small group differences. We used a Bonferroni-adjusted *p*-value significance cut-off of *p* < 0.05. Secondly, we used beta values transformed to M-values (M_CpG i_=log2(beta_CpG i_/(1-beta_CpG i_))) as M-values have been proposed to be more appropriate for statistical testing, although more challenging to interpret^48^. For this analysis, we used the same type of test (Wilcoxon’s test) and significance cut-off (Bonferroni-adjusted *p* < 0.05) complemented by a similar requirement of a minimum absolute group difference (abs(beta)>0.25 equivalent to abs(M) > 1.584963). In addition, to avoid infinite M-values caused by the log2 transformation we capped beta values at 0.999 and 0.001.

CpGs used in supervised and unsupervised analyses were annotated as outlined by Aine et al.^45^. Briefly, we compiled a custom feature annotation set for each CpG probe on the Illumina EPIC methylation platform using the same methodology described for the Illumina HumanMethylation450K array in Staaf and Aine^46^. This included assigning CpGs to a gene-centric context defined as promoter (+/− 500 bp centered on gene transcription start site, TSS), proximal (+/− 5 kbp centered on TSS and excluding the promoter window), or distal (>5 kbp from TSS) based on their genomic coordinates (referred to as genic context). For the gene-centric annotations, a consensus transcript model based on GENCODE (v27) protein coding genes matching SCAN-B RNA-sequencing data was built for each gene by collapsing of exons. The 5’ most base was assigned as the consensus TSS and the 3’ most base as the consensus transcription termination site. Probes were also assigned to a CpG-centric context defined as CpG island (CGI), shore, or ocean (referred to as CGI context)^49^. Local CpG density metrics (e.g., observed/expected, O/E) and contextual classifications for each probe were obtained using the methods of Saxonov et al.^50^ for high (HCG) and low (LCG) CpG content and of Weber et al.^51^ for HCP (high), ICP (intermediate), and LCP (low). EPIC probe overlaps with ATAC-seq peak data generated on 74 TCGA breast cancer samples by Corces et al.^52^ were calculated and used as a proxy for differentially open chromatin in breast cancer. Additionally, ENCODE candidate cis-regulatory elements^53^ and ENCODE ChIP-seq peak overlaps for 340 transcription factors in 130 cell lines^54^ were used to assess the regulatory potential of EPIC CpGs.

### Survival analyses

Survival analyses were performed in the final Endo and ChemoEndo treatment groups. For the Endo group, cancer registry outcome data from ref. ^27^. were used (due to incomplete available clinical review of all cases), whereas for the ChemoEndo group, clinical review data were available for the 339 patients. DRFI, defined according to the STEEP criteria^55^, and OS were used as primary endpoints for both treatment groups. Median outcome measures in censored patients in the ChemoEndo cohort were equal to 7.2 and 8.6 years for DRFI and OS, respectively, and 6.5 and 8.4 years in the Endo cohort for DRFI and OS, respectively. Survival analyses were performed in R (v4.4.2) using the *survival* (v3.7.0) and *survminer* (v0.5.0) packages. Survival curves were estimated using the Kaplan-Meier method and compared using the log-rank test. Hazard ratios were calculated through univariable and multivariable Cox regression using the *coxph* R function. A *p*-value < 0.05 was considered significant.

### Statistics and reproducibility

All patients from the reported SCAN-B study who fit the defined breast cancer subgroups were included, with a subset analyzed by WGS and DNA methylation profiling as outlined in the study’s CONSORT figure (Supplementary Fig. S1a). No replication of WGS, RNA-sequencing or DNA methylation analyses of tumors was performed. Neither randomization nor blinding were applicable to this study. This study is based on groups defined by clinicopathological variables or molecular variables (like PAM50 subtypes), for which statistical comparisons were performed, with the statistical tests reported together with the exact *p*-values. All *p*-values reported from statistical tests are two-sided if not otherwise specified. Upset plots were created using the UpSetR package (v1.4.0). All cohorts used in this study are retrospective.

## Results

### HRD frequencies and relationship with transcriptional subtypes

We first evaluated the population representativeness of our WGS-analyzed primary SCAN-B ERpHER2n BC cohort (with no patients receiving treatment prior to tissue sampling). Indeed, ChemoEndo cases in our study were comparable to ChemoEndo patients in the complete catchment region for the inclusion years, while the Endo group in our study was skewed towards a more aggressive phenotype, as shown by higher proportions of grade 3 tumors and more proliferative tumors according to the Ki67 marker, consistent with the study’s patient selection criteria and layout (see Supplementary Fig. S1a–d).

**Fig. 1:**
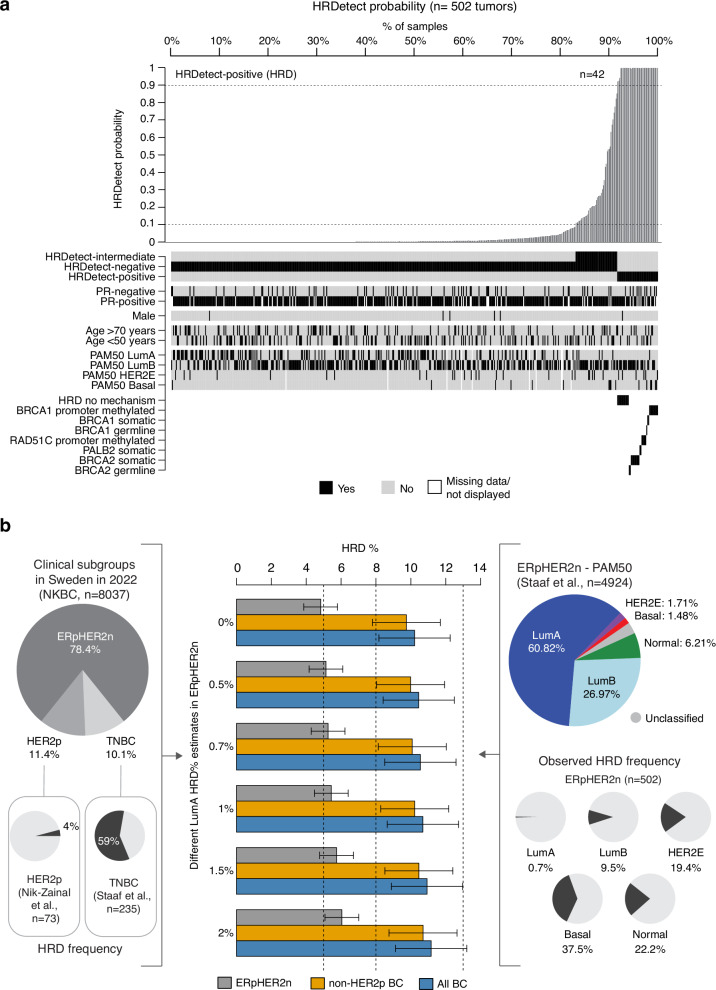
HRD in ERpHER2n. **a** HRDetect probability for 502 WGS analyzed ERpHER2n SCAN-B tumors and their characteristics. HRD is defined as an HRDetect probability ≥0.9. **b** Estimation of HRD frequency in breast cancer for ERpHER2n tumors, non-HER2-positive tumors (non-HER2p), and all breast cancer (including ERpHER2n, HER2-positive, and TNBC tumors). Estimations are based on the clinical subgroup proportions reported to the national Swedish breast cancer registry (NKBC) in 2022 (left pie chart), the reported PAM50 subtype proportions in ERpHER2n tumors from Staaf et al.^27^ (right pie chart), HRD frequency in population-based TNBC^9^, HRD frequency in HER2-positive disease^6^, and the estimated HRD frequency in PAM50 Basal, HER2E, LumB, and Normal subtypes in this study. Final estimates are presented as a range based on calculations using different assumed HRD frequencies in ERpHER2n LumA tumors. Error bars represent the span of estimated HRD frequency based on the assumption of 20% error in the individual HRD point estimates.

Of the 502 patients, 42 (8.4%) were classified as HRD (HRDetect score ≥0.9, otherwise HR-proficient), with an HRD frequency of 7.2% in the Endo and 9.1% in the ChemoEndo treatment group (Fig. 1a, Table 1, Supplementary Data 2). Based on matched germline screening information, 57 of the 502 patients had undergone germline screening for a set of different genes (see ref. ^28^ for details) (Table 1). Of the 42 patients with HRD tumors, three (7.1%) had a previously known germline pathogenic variant in *BRCA1/BRCA2*, three (7.1%) had somatic homozygous *BRCA1*/*BRCA2* deletions, and 10 (23.8%) had only somatic pathogenic variants, indels, or structural rearrangements affecting *BRCA1*, *BRCA2*, or *PALB2* detected by WGS. An HRDetect-intermediate classification (score ≥0.1 and <0.9) was observed in 8.6% of patients. Patients with HRD tumors showed no significant difference in age at diagnosis, tumor size, HER2-low status (HER2 IHC 2+/ISH- or IHC 1+), or lymph node status compared to HR-proficient cases for the total cohort, the ChemoEndo subset, or the Endo subset (two-sided *p* > 0.05, Wilcoxon’s test or Chi-square test). HRD tumors showed higher Ki67 index, lower levels of ER and PR-stained cells, and higher tumor grade (nonsignificant trend in Endo subset) compared to other tumors in the total cohort, ChemoEndo, and/or Endo subsets (Supplementary Fig. S2). Two tumors (0.4%) showed signatures of mismatch repair deficiency (MMRd). Both were scored as HR-proficient by HRDetect; however, one case (S002375), carrying a somatic biallelic *BRAC2* mutation, appears to have a mixed HRD/MMRd phenotype. Finally, six of the 502 patients were male, only one with a tumor classified as HRD (16.7%).

We next assessed the relationship between HRD in ERpHER2n tumors and PAM50 subtypes, a common BC stratification strategy based on gene expression. HRD frequencies were 37.5% in the PAM50 Basal subtype (*n* = 6/16), 19.4% in HER2E (6/31), 0.7% in LumA (1/152), 9.5% in LumB (27/284) tumors, and 22.2% in Normal (2/9) tumors. Thus, HRD can occur across all PAM50 subtypes, albeit with different frequencies.

### Population frequency of HRD in ERpHER2n BC

For each PAM50 subtype, we further contrasted patient age, tumor size, lymph node status, tumor grade, and gene expression rank scores of five biological metagenes (representing immune response, steroid response, basal expression, and proliferation)^28,32^ between our WGS cohort versus an independent set of 4427 non-WGS ERpHER2n tumors of unknown HRD status^27^. These analyses demonstrated that the PAM50 Basal, HER2E, and LumB subgroups in the WGS cohort showed similar characteristics to non-WGS profiled cases and may be viewed as population-representative, whereas PAM50 LumA and Normal tumors in this WGS cohort cannot be considered population-representative (Supplementary Fig. S3).

Based on these results, we estimate the prevalence of HRD in ERpHER2n BC and BC as a whole by combining our estimates with HRD estimates in TNBC and HER2-positive tumors from previous publications^6,9^. Specifically, we merged our HRD estimations in the PAM50 Basal, HER2E, LumB, and Normal subtypes with additional population-representative BC datasets reporting PAM50 subtype proportions in ERpHER2n tumors (*n* = 4924 tumors)^27^, HRD frequency in TNBC (59%)^9^, HRD frequency in HER2-positive tumors^6^ (4.1%, 4/73 WGS and HRDetect analyzed cases), and data from the Swedish National Breast Cancer Quality Registry (NKBC) concerning proportions of TNBC, HER2-positive, and ERpHER2n disease in the total Swedish population in 2022 (Fig. 1b). Combining HRD and PAM50 subtype proportions for ERpHER2n tumors, we infer an HRD frequency of 4.8% in ERpHER2n BC (excluding the contribution from LumA or unclassified tumors). Next, combining the ERpHER2n HRD estimate with NKBC clinical subtype proportions and our HRD estimates in TNBC and HER2-positive tumors, we can model an overall HRD frequency that varies depending on assumptions of HRD proportions in ERpHER2n LumA tumors (excluding unknown contributions from the small set of PAM50 unclassified ERpHER2n tumors in ref. ^27^) For instance, with the observed HRD frequency of 0.7% in ERpHER2n LumA tumors, the final overall HRD frequency would be 10.1%, or 1 in 10 in HER2-negative BC patients, or 10.5% in all breast cancer (TNBC, HER2-positive, and ERpHER2n combined).

### Agreement in HRD classifications by alternative methods in ERpHER2n BC

**Fig. 2:**
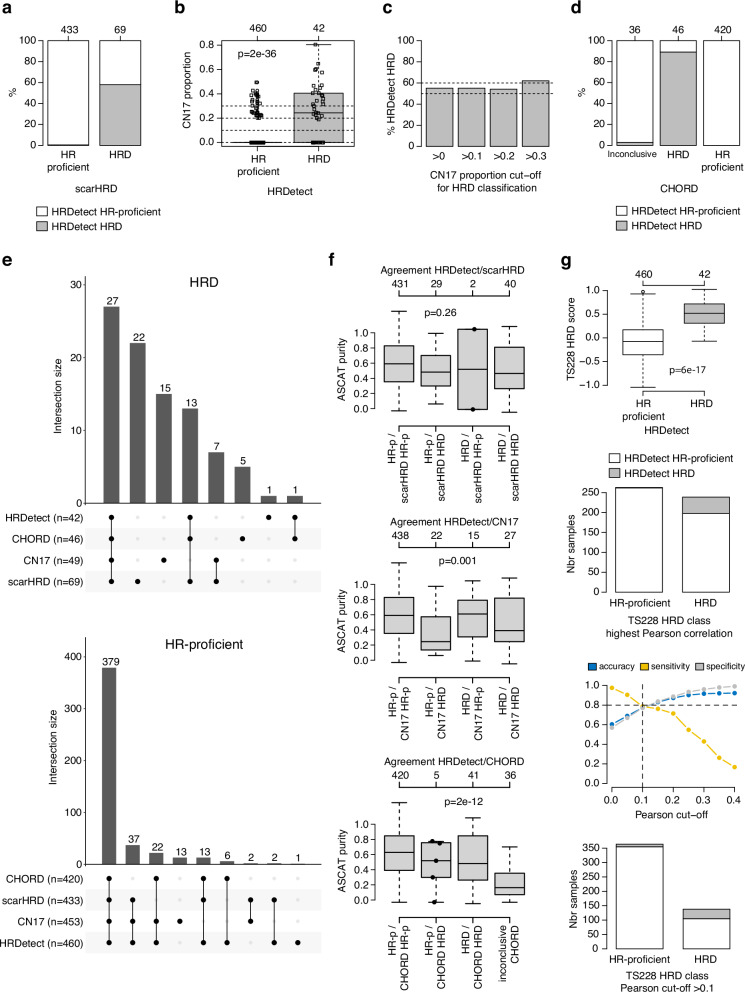
Comparison of DNA-based HRD classification methods in ERpHER2n BC. **a** HRDetect status for tumors classified as scarHRD HR-proficient or HRD. **b** CN17 signature proportions versus HRDetect status. Two-sided *p*-values calculated using Wilcoxon’s test. **c** Proportion of HRDetect HRD tumors in groups of CN17 HRD tumors classified using different cut-offs for the CN17 signature proportion. **d** HRDetect status for tumors classified as CHORD HR-proficient, HRD, or undetermined. **e** Upset plots of classification overlap between HRD methods. For CN17 a cut-off of >0 was used to call HRD. For CHORD, 36 tumors could not be classified and are not included. **f** ASCAT WGS estimated tumor purities versus concordant and discordant HRD status between HRDetect and the other methods. HR-p: HR-proficient. For CN17, a cut-off of >0 was used to call HRD. Two-sided *p*-values calculated using the Kruskal-Wallis test. Individual data points are shown for groups ≤5 tumors in size. **g** Results of the TS228 gene expression HRD classifier versus HRDetect in the 502 tumors. Panels show from top to bottom: (i) distribution of an expression-based HRD score versus HRDetect status with two-sided *p*-value calculated using Wilcoxon’s test, (ii) composition of HRDetect HRD status in HRD groups defined by gene expression using the highest centroid correlation only, (iii) accuracy, sensitivity, and specificity for gene expression vs HRDetect classification using different Pearson correlation cut-offs applied to the HRD centroid, and (iv) composition of HRDetect HRD status in HRD groups defined by gene expression using an optimized Pearson cut-off of 0.1. Boxplot elements correspond to: (i) center line = median, (ii) box limits = upper and lower quartiles, (iii) whiskers = 1.5x interquartile range. In boxplots and barplots, top axes indicate group sizes.

HRD status can be predicted in multiple ways using DNA-based methods^7,17,18^. To assess the agreement between different DNA-based methods we compared HRD classification by HRDetect versus HRD status called by CHORD^24^, scarHRD (representing an HRD score involving telomeric allelic imbalances, loss of heterozygosity profiles, and large-scale state transitions)^41^, and a copy number signature approach (based on the CN17 signature proposed to be associated with HRD by Steele et al.^40^). For scarHRD, we found a concordance of 0.94 in HRD status compared to HRDetect; however, only about 60% of scarHRD HRD tumors were HRD by HRDetect resulting in a lower positive predictive value (PPV = 0.58) (Fig. 2a). Similarly, while CN17 proportions were significantly associated with HRDetect status (Fig. 2b), thresholded classification of CN17 proportions showed that about 55-60% of CN17 HRD cases were HRD by HRDetect (Fig. 2c). For the sequencing-based CHORD method, 466 of the 502 tumors could be classified (92.8%). Agreement between CHORD and HRDetect for these 466 tumors was 0.99, while the PPV was 0.89 using HRDetect as reference (Fig. 2d). Upset plots for HRD and HR-proficient tumors revealed that 27 of the 42 (64.3%) HRDetect HRD tumors were classified as HRD by all four methods, while 82.4% (379/460) of HRDetect HR-proficient tumors were classified as HR-proficient by all methods (Fig. 2e).

To assess whether discordance between HRDetect and the other DNA-based methods was due to tumor cellularity, we compared ASCAT tumor purity estimates versus combined classifications. This analysis showed that discordance for scarHRD did not seem associated with tumor purity, while tumors that could not be classified by CHORD, as well as false positive CN17 tumors (HRDetect HR-proficient but CN17 HRD), appeared to have lower tumor cell content (Fig. 2f).

Finally, we also compared HRDetect status to HRD status based on a gene expression-based nearest centroid HRD classifier (TS228) reported by Jacobson et al.^36^ for the 502 tumors (Fig. 2g). While the TS228 gene expression HRD score was significantly different between HRDetect HRD and HR-proficient tumors, classification using the highest centroid correlation resulted in a substantial number of HRDetect HR-proficient tumors being called as HRD. Instead, analysis of a range of correlation cut-off values suggested that 0.1 was the optimal Pearson correlation cut-off to the HRD centroid (Fig. 2g). This cut-off resulted in an accuracy, sensitivity, and specificity of HRD for TS228 of approximately 80% compared to HRDetect, in line with the original study results^36^. Still, this optimized cut-off resulted in a substantial number of HRDetect HR-proficient tumors being called as HRD by the gene expression classifier (Fig. 2g).

### Transcriptional features of HR-deficient ERpHER2n BC are diverse

**Fig. 3:**
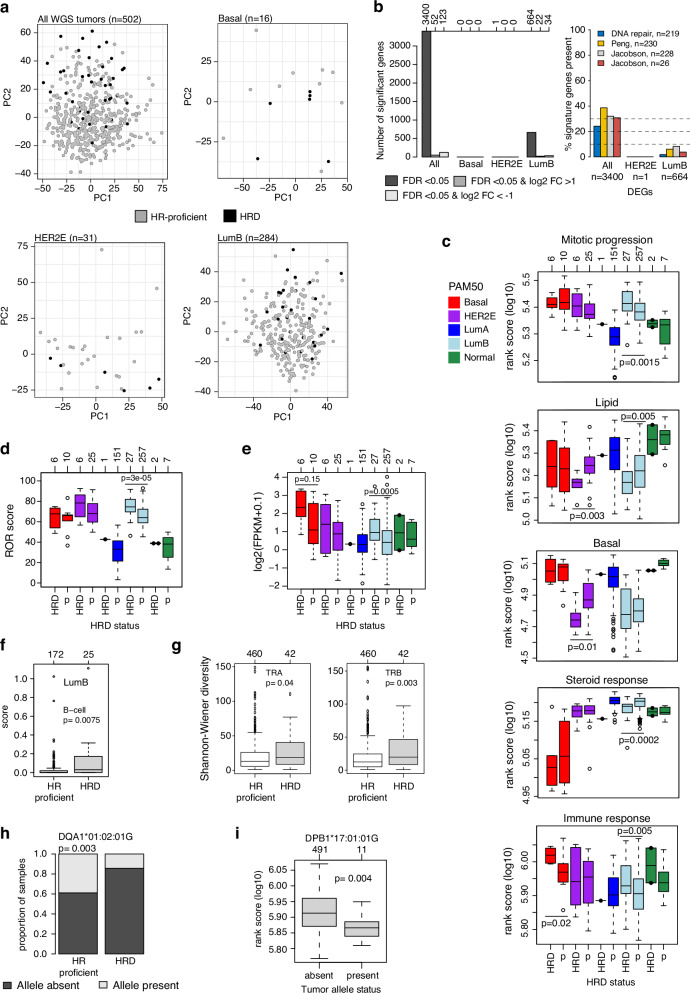
Transcriptional patterns in ERpHER2n BC with respect to HRD status. **a** Principal component analysis in all ERpHER2n tumors and in each PAM50 subtype using the 5000 most variant genes based on FPKM data for the respective group. HRD tumors are marked by black points. Principal components 1 and 2 (PC1 and PC2, respectively) are shown. The LumA group is excluded due to only one HRD tumor. **b** Left: summary of the number of differentially expressed genes per PAM50 subtype based on different FDR-adjusted two-sided *p*-values and log2 fold-changes (FC). The top axis indicates the exact number of genes for each bar. Right: proportion of gene overlap between four gene signatures and the differentially expressed gene sets for all tumors, HER2E, and LumB tumors. **c** Gene expression rank scores (log10-transformed) for five biological metagenes defined by Fredlund et al.^32^. stratified by PAM50 subtype and HRD status (p = HR-proficient). Only significant associations are shown as two-sided *p*-values, calculated using Wilcoxon’s test. The top axis indicates group sizes. **d** ROR-score as calculated by Staaf et al.^27^. Two-sided *p*-values calculated using Wilcoxon’s test. *p* = HR-proficient. **e** mRNA expression of PD-L1 (*CD274*) versus HRD status in PAM50 subtypes. Only significant or borderline nonsignificant associations are shown as two-sided *p*-values, calculated using Wilcoxon’s test. p = HR-proficient. **f** CIBERSORTx estimates for B-cells in LumB tumors stratified by HRD status. Two-sided *p*-value calculated using Wilcoxon’s test. Not all tumors have values based on CIBERSORTx *p*-value filtering. **g** Shannon-Wiener diversity scores computed from bulk tumor RNA-sequencing data in all tumors for the T-cell receptor genes *TRA* and *TRB* stratified by HRD status. Two-sided *p*-values calculated using Wilcoxon’s test. **h** Proportion of HR-proficient and HRD tumors with the DQA1*01:02:01 G allele present or absent. Two-sided *p*-value calculated using the Chi-square test. **i** Log10 immune rank scores versus present/absent allele status for the DPB1*17:01:01G allele. Two-sided *p*-value calculated using Wilcoxon’s test. Boxplot elements correspond to: (i) center line = median, (ii) box limits = upper and lower quartiles, (iii) whiskers = 1.5x interquartile range. In boxplots, top axes indicate group sizes. In **c**–**e**, individual data points are shown for groups ≤5 tumors in size.

HRD tumors have reported selective therapeutic vulnerabilities, raising the question of whether transcriptional profiling could identify HRD for precision medicine purposes. We thus investigated whether ERpHER2n HRD tumors showed consistency in transcriptional profiles across PAM50 subtypes, limiting our analyses to the Basal, HER2E, and LumB PAM50 subtypes, where we were powered and more population representative. First, we performed unsupervised gene expression analysis using PCA, contrasting cases with and without HRD. Variance within tested groups using 5000 of the most variable genes, whether across the whole cohort or separated by PAM50 subtypes, could not be explained by HRD status (Fig. 3a). Similar results were observed using different gene set cut-offs (Supplementary Fig. S4). Thus, HRD status is not singularly a major determinant of transcriptional variation in ERpHER2n disease.

Second, performing a supervised differentially expressed gene (DEG) analysis contrasting HRD status in the whole cohort, we found that 3400 genes differed between HRD and HR-proficient tumors. A GSEA of this gene set using hallmark signatures from MSigDB^56^ identified 10 significant hallmarks related to proliferation (checkpoint and mitotic spindle), immune response (interferon response, allograft rejection), estrogen signaling, MTORC1 signaling, and MYC and E2F targets (Supplementary Data 3). Notably, these hallmark enrichments are highly consistent with findings by Ballot et al.^22^. However, when a supervised approach was applied within the Basal, HER2E, and LumB PAM50 subtypes, no DEGs were detected in Basal, and only one gene in HER2E (*BAG5*). It should be acknowledged that the lack of significant DEGs in Basal and HER2E tumors may be caused by the small group sizes, combined with multiple testing correction. In LumB, 664 genes were differentially expressed (FDR-adjusted two-sided *p* < 0.05), however, with typically small fold-changes for the majority of genes. GSEA analysis of this gene set identified only three KEGG (Kyoto Encyclopedia of Genes and Genomes) pathways that appeared unrelated: Epstein-Barr virus infection, Prion disease, and Lysine degradation (Fig. 3b, Supplementary Fig. S4, Supplementary Data 3). DEGs were also compared to a list of 219 DNA repair-associated genes and three HRD-associated gene lists (Peng et al., *n* = 230^57^, and Jacobson et al., *n* = 228 and *n* = 26^36^, listed in Supplementary Data 4). Notably, within the 3400 DEGs identified in the all tumor group comparison, 24–39% of genes in the four gene lists were represented; however, in the specific LumB group comparison with 664 DEGs, the gene representation declined substantially (2–8%) (Fig. 3b, Supplementary Data 3).

We computed rank scores for eight gene sets (or metagenes) representative of different biological processes (stroma, immune response, lipid, mitotic checkpoint, mitotic progression, basal expression, steroid response, and early response genes)^32^ for each tumor to elucidate differences in large-scale transcriptional profiles between HRD and HR-proficient tumors. In addition, an RNA-sequencing-based ROR score, developed as part of the Prosigna prognostic BC assay^58^, was also computed as described^27^. In LumB, HRD tumors were associated with higher rank scores for the immune response metagene and proliferation-associated metagenes connected to mitotic progression, while showing lower rank scores for the steroid response metagene, consistent with lower levels of ER and PR-stained tumor cells (Fig. 3c). Higher tumor proliferation observed in HRD compared to HR-proficient LumB tumors was reinforced by both higher ROR scores and Ki67 IHC indices (Fig. 3d, Supplementary Fig. S4). In PAM50 Basal tumors, HRD tumors showed higher immune response metagene rank scores compared to HR-proficient tumors, but there was no difference in proliferation or steroid response metagenes (Fig. 3c). In the HER2E subtype, HRD tumors showed a non-significant trend towards higher proliferation, while significantly lower expression of the basal and lipid metagenes (Fig. 3c). Overall, HRD status in ERpHER2n disease is not represented by a unique transcriptional profile. HRD tumors exhibit diverse transcriptional profiles with evidence of higher tumor proliferation and/or immunologic potential in some, but not all, PAM50 subtypes.

### Specific immune features of HR-deficient ERpHER2n BC

Although immune infiltration estimates based on RNA-sequencing should ideally be validated in situ, for instance through estimation of tumor infiltrating lymphocytes (TILs), previous findings in SCAN-B TNBC tumors have demonstrated that the specific immune expression metagene used above shows high correlation to whole-slide hematoxylin and eosin (H&E) TIL counts based on pathologist scoring^59^. To further investigate immune response differences between HRD and HR-proficient tumors, we analyzed programmed cell death-ligand 1 (PD-L1) mRNA expression (*CD274*) and immune cell types based on CIBERSORTx-deconvoluted RNA-sequencing data. Higher *PD-L1* expression was observed in mainly Basal (trend-like) and LumB (significant) HRD tumors, consistent with the immune metagene patterns (Fig. 3e). In LumB tumors (the subtype with most cases), B-cells were the only significant CIBERSORTx cell type (of B-cells, CD8 T-cells, CD4 T-cells, NK-cells, monocytes, and neutrophils) associated with HR status (Fig. 3f). We also analyzed associations between T-cell/B-cell receptor diversity, based on computed RNA-sequencing scores, and HRD status. Focusing on the T-cell receptor genes *TRA* and *TRB* (due to read count levels), we observed a higher Shannon-Wiener diversity in HRD tumors compared to HR-proficient tumors for both T-cell receptor genes (Fig. 3g), correlating with the higher immune metagene scores. No statistically significant differences were observed for the analyzed B-cell receptor genes *IGH*, *IGK*, and *IGL* (two-sided Wilcoxon’s test *p* > 0.05).

Finally, we also analyzed HLA status based on DNA copy number levels using HLA*LA-HLA typing^43^. HLA*LA-HLA provided data on 13 different loci and 191 unique alleles across the 502 tumors. There was no locus with statistically different proportions (defined as present/absent) between HRD and HR-proficient tumors. While four unique alleles showed different proportions of presence/absence between HRD and HR-proficient tumors (two-sided Chi-square test *p* < 0.05, example shown in Fig. 3h), these differences were not significant after adjusting for multiple testing (FDR adjusted two-sided *p* > 0.05). We also compared specific allele status versus the immune expression metagene rank scores, finding only three unique alleles with a two-sided Wilcoxon’s *p* < 0.05 that did not remain significant after adjustment for multiple testing (FDR adjusted two-sided *p* > 0.05, example in Fig. 3i).

### Genomic features of HR-deficient ERpHER2n BC

**Fig. 4:**
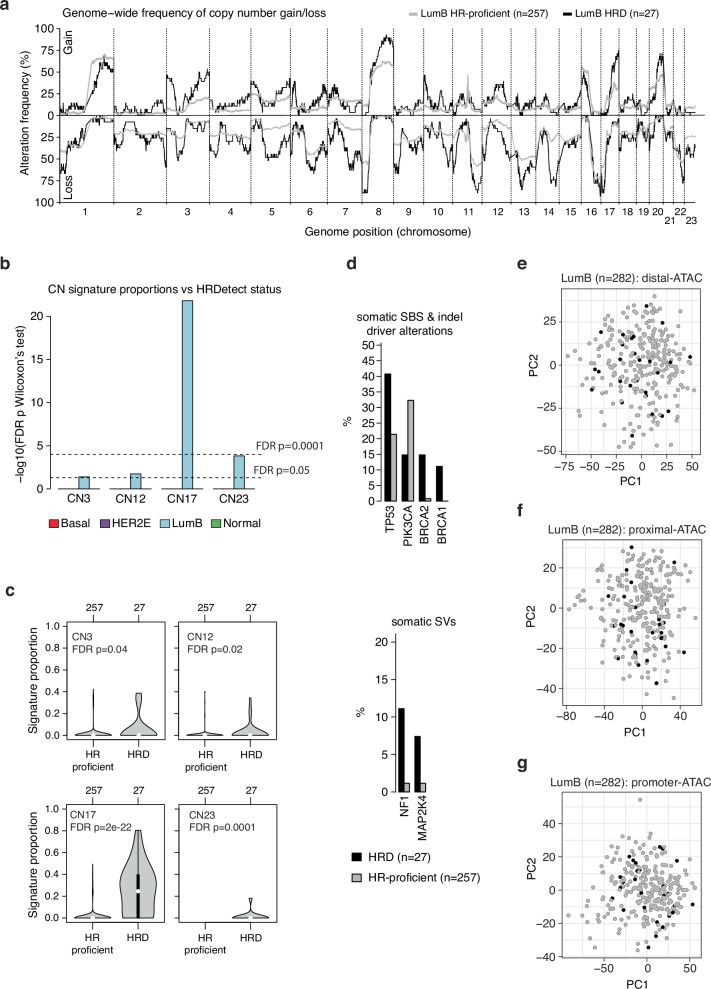
DNA alterations and DNA methylation patterns in ERpHER2n BC with respect to HRD status. **a** Genome-wide frequency of copy number gain and loss in LumB tumors stratified by HRDetect status. **b** Bar plot of FDR-adjusted two-sided Wilcoxon’s test values (-log10 transformed) for four copy number signatures (CN) with an FDR-adjusted *p* < 0.05 between HR-proficient and HRD tumors stratified by PAM50 subtype. **c** Proportions of CN3, CN12, CN17, and CN23 in LumB tumors stratified by HRD status. Two-sided *p*-values were calculated using Wilcoxon’s test and adjusted for FDR. **d** Frequency of driver alterations in LumB tumors differs between HRD and HR-proficient tumors. Only genes with at least two affected tumors and a Fisher’s exact test two-sided *p* < 0.1 are shown. **e** Principal component analysis of tumor purity-adjusted DNA methylation data (beta values) based on the 5000 most variant distal-ATAC CpGs in LumB tumors. Black dots represent tumors with an HRDetect HRD classification. The first two principal components (PC1 and PC2) are shown. **f** Same as in E, but for CpGs in a proximal-ATAC context. **g** Same as in (**e**), but for CpGs in a promoter-ATAC CpG context. Boxplot elements correspond to: (i) center line = median, (ii) box limits = upper and lower quartiles, (iii) whiskers = 1.5x interquartile range. In violin plots, top axes indicate group sizes.

We next contrasted genomic features of HRD and HR-proficient tumors within PAM50 subtypes. A variety of features were evaluated, including genome-wide patterns of copy number alterations (CNAs), the fraction of the genome altered by CNAs and loss of heterozygosity (LOH), tumor mutational load, tumor ploidy, mutational and rearrangement signatures (Supplementary Fig. S5). HRD tumors showed higher total mutational load and genomic instability distributed through the genome (SBSs, indels, and/or SVs) compared to HR-proficient tumors, irrespective of PAM50 subtype. HRD tumors did not have statistically different tumor ploidy compared to HR-proficient cases but did show more regions of copy number gain or loss and generalized LOH compared to HR-proficient tumors in the total cohort, LumB, and HER2E tumors (as exemplified for copy number alterations in LumB tumors in Fig. 4a and furthermore in Supplementary Fig. S5). We also compared proportions of 25 copy number signatures^40^ in tumors stratified by HRDetect and PAM50 status (Supplementary Fig. S5). Similar to the subtype-specific mRNA analyses, this analysis is also statistically limited by lower sample numbers in Basal, HER2E, and Normal tumors, and correction for multiple testing. In agreement with an HRD phenotype, elevated proportions of CN17 (associated with HRD^40^) were observed in HRD LumB tumors, while several other statistically significant signature differences in LumB tumors (like CN3, CN12, and CN23) appeared less distinct and likely outlier-driven (Fig. 4b, c). Combined, these analyses demonstrate that HRD ERpHER2n tumors typically have more complex genomes compared to HR-proficient tumors across PAM50 subtypes. The relative frequency of somatic driver events (SBSs, indels, and SVs) in HRD and HR-proficient tumors was also investigated (Supplementary Fig. S5). The relative proportion of *TP53* drivers was higher in the HRD tumors in all subtypes (example for LumB in Fig. 4d), and a lower *PIK3CA* mutation rate in HRD LumB tumors compared to HR-proficient LumB was observed, in line with previous findings (Fig. 4d)^22,36^.

### Global DNA methylation features of HR-deficient ERpHER2n BC

To investigate if HRD tumors display different global DNA methylation patterns compared to HR-proficient tumors we performed a PCA of tumor purity-adjusted CpG beta values (representing conceptually purer tumor methylomes, see ref. ^46^) obtained from Illumina EPIC DNA methylation arrays in the total cohort (*n* = 499), and restricted to the PAM50 subtypes Basal (*n* = 16), HER2E (*n* = 31), and LumB (*n* = 282) due to sample numbers. PCAs were performed in three different CpG contexts (gene distal, promoter proximal, and promoter) to acknowledge CpG density differences in the genome. Each CpG set was further filtered to only include the 5000 most variant CpGs mapping to proposed regions of open chromatin defined by ATAC-sequencing (obtained from ref. ^52^ distal-ATAC, proximal-ATAC, and promoter-ATAC), as this was recently shown to define more information rich CpG subsets in TNBC^45^. Notably, in all PCA comparisons, HRD status did not appear as a distinct divider of variation (Fig. 4e–g showing LumB and Supplementary Fig. S5 for all results). Next, we performed differential DNA methylation analyses between HRD and HR-proficient tumors in the same sample groups for all 741144 CpGs. Only in all tumors and LumB tumors were significantly differentially methylated CpGs identified, but in very small numbers after multiple testing adjustment (*n* = 55 and 4, respectively, two-sided Bonferroni-adjusted Wilcoxon’s test *p* < 0.05, and mean absolute difference in beta > 0.25 between groups). To further explore differential methylation between HRD and HR-proficient tumors, we performed a similar analysis using M-values instead of beta values, as the former has been proposed as a better alternative for differential methylation analysis^48^. Using M-values, we found 911 significant CpGs in the HRD/HR-proficient comparison, including all tumors, 35 CpGs in LumB tumors, and no CpGs in Basal or HER2E tumors. It should be acknowledged that the lack of significant CpGs in Basal and HER2E tumors may be caused by the small group sizes, combined with multiple testing correction. Together, the unsupervised and supervised DNA methylation analyses suggest that HRD status is not associated with specific or distinct global DNA methylation patterns in ERpHER2n tumors.

### Mechanisms of HRD in ERpHER2n BC

To understand gene inactivation mechanisms underpinning the 42 HRD ERpHER2n tumors, we examined known germline status, somatic variants, and DNA methylation status of promoter CpGs for 219 DNA repair deficiency-associated genes (listed in Supplementary Data 4). Thirty (71.4%) cases had potentially causative genetic/epigenetic events involving *BRCA1*, *BRCA2*, *RAD51C*, or *PALB2*, while 28.6% (12/42) of cases lacked a causally implicated event (Fig. 1a, Supplementary Data 5). Among those with a potentially causative event, promoter hypermethylation of *BRCA1*/*RAD51C* accounted for ∼47%, WGS-based somatic alterations (SNVs, indels, structural rearrangements, and homozygous deletions) underpinned ∼43%, and pathogenic germline variants explained 10% of HRD cases. Importantly, clinical germline screening data were not available for all HRD patients in this study due to privacy restrictions, preventing full review of germline data in SCAN-B patients. Consequently, the impact of pathogenic germline variants as an inactivation mechanism is likely underestimated in this cohort.

**Fig. 5:**
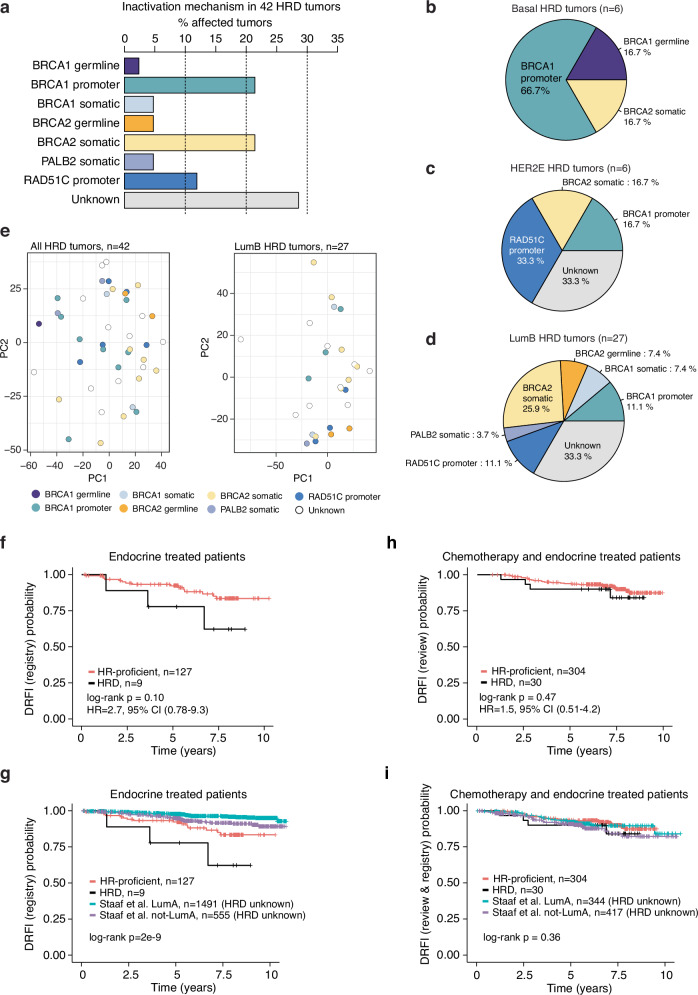
Inactivation mechanisms in HRD tumors and associations with patient outcome. **a** All 42 HRD tumors. **b** Six HRD PAM50 Basal tumors. **c** Six HRD PAM50 HER2E tumors. **d** 27 HRD PAM50 LumB tumors. In **a**–**d**, a few cases exist harboring both promoter methylation and a somatic variant for a gene. For these cases, only one of the alterations has been counted as detailed in Supplementary Data 5, based, e.g., on correlation to mRNA expression. **e** Principal component analysis in all HRD tumors (left) and in LumB HRD tumors (right) using the 5000 most variant genes based on FPKM data for the respective group, colored by the proposed HRD inactivation mechanism. Principal components 1 and 2 (PC1 and PC2, respectively) are shown. The LumA, Basal, and HER2E groups are excluded due to a few cases. **f** Kaplan-Meier plot of the association of HRD status with DRFI for patients with HRD and HR-proficient WGS analyzed tumors treated with Endo. DRFI was based on cancer registry data from Staaf et al.^27^. Univariate Cox regression hazard ratio and 95% confidence interval shown. **g** Kaplan-Meier plot of the association of HRD status with DRFI for HRD and HR-proficient WGS analyzed tumors with inclusion of 2046 additional non-overlapping Endo-treated patients with unknown HRD status from Staaf et al.^27^ stratified by their PAM50 LumA status (LumA or not-LumA). DRFI was based on cancer registry data from ref. ^27^ for non-WGS patients. **h** Kaplan-Meier plot of the association of HRD status with DRFI for patients with HRD and HR-proficient WGS analyzed tumors treated with ChemoEndo. Clinical review: DRFI data were used as the endpoint. Univariate Cox regression hazard ratio and 95% confidence interval shown. **i** Kaplan-Meier plot of the association of HRD status with DRFI for HRD and HR-proficient WGS analyzed tumors with inclusion of 761 non-overlapping ChemoEndo treated patients with unknown HRD status from Staaf et al. ^27^ stratified by their PAM50 LumA status (LumA or not-LumA). For the 761 patients, the DRFI data were based on cancer registry data from ref. ^27^, while for the WGS analyzed samples, clinical review data were used as the endpoint. In Kaplan-Meier plots, the *p*-value was calculated using the log-rank test.

*BRCA1* and *RAD51C* promoter hypermethylation were observed in 21.4% (9/42) and 11.9% (5/42) of HRD tumors, respectively (Fig. 5a), with biallelic inactivation through LOH or a somatic variant observed in 92.8% (13/14) of these hypermethylated cases. Promoter hypermethylation of *BRCA1* and *RAD51C* was also frequent within PAM50 subtypes, with 66.7% *BRCA1* promoter methylation frequency alone in PAM50 Basal HRD tumors, 50% frequency of combined *BRCA1* and *RAD51C* hypermethylation in HER2E HRD tumors, and 22% combined frequency in PAM50 LumB HRD tumors (Fig. 5b–d). We have previously shown that TNBC tumors with inactivated *RAD51C* and *PALB2* present a genetic phenotype similar to *BRCA2*-deficient tumors^9^. To examine whether the type of inactivation mechanism was associated with distinct transcriptional patterns in HRD tumors, we performed PCAs for all 42 HRD tumors and for the 27 LumB HRD tumors specifically (due to numbers). However, we did not find that tumors with *BRCA2*/*PALB2*/*RAD51C* alterations appeared largely different from *BRCA1* inactivated cases (Fig. 5e). Substantiating this finding, we reached a similar conclusion regarding inactivation mechanism and global DNA methylation variation using PCA performed as above in LumB HRD tumors specifically (Supplementary Fig. S5).

Regarding other DNA repair deficiency genes, two patients had clinically reported pathogenic germline variants in *ATM*, and one had a pathogenic germline variant in *CHEK2*. However, all tumors showed an intact alternative allele and were HR-proficient. No tumor showed promoter hypermethylation of *ATM* or *CHEK2*. Two HR-proficient tumors had somatic *CHEK2* variants, while seven tumors had somatic *ATM* SBS or indel drivers. Only one of these tumors was HRD (without any variants in *BRCA1*, *BRCA2*, *PALB2*, or *RAD51C*). Together, these data do not support *ATM* or *CHEK2* alterations as important mechanisms for HRD in ERpHER2n BC.

### Association of HRD with prognosis in ERpHER2n BC

Finally, to understand the prognostic value of HRD status in ERpHER2n cancers treated with Endo and ChemoEndo, we assessed the association between HRD status and DRFI. In the non-representative Endo WGS cohort (*n* = 136), HRD status was borderline nonsignificantly associated with differences in DRFI (log-rank *p* = 0.10, Fig. 5f), although it needs to be stressed that the cohort and especially the number of HRD cases are small. In a multivariate Cox regression analysis including tumor size (mm), nodal status (N0/N+), and tumor grade as covariates, and DRFI as clinical endpoint, HRD status was not significant in the Endo cohort. Because of these limitations, we included an independent non-overlapping cohort of 2,046 Endo-only patients with unknown HRD status^27^ to contrast outcome patterns. Notably, the HRD group showed the poorest outcome of all groups despite its sample size, and LumA cases exhibited the best outcome (Fig. 5g). Supporting the skewness of our HR-proficient WGS Endo cases, these patients had a borderline nonsignificant trend of poorer DRFI compared to patients with non-LumA tumors in the independent cohort (log-rank *p* = 0.06). Thus, although not population-representative and hampered by low numbers, we observe a trend that warrants further consideration – patients with ERpHER2n HRD tumors are unlikely to have good outcomes on Endo treatment alone and may benefit from additional or alternative therapeutic strategies.

In the population-representative ChemoEndo cohort, HR status was not associated with differences in DRFI (log-rank *p* = 0.47, Fig. 5h), although a nonsignificant trend towards poorer overall survival was observed in HRD patients (log-rank *p* = 0.08). The observation of a nonsignificant association of HRD status with outcome in the ChemoEndo group holds true regardless of the HRD assay used (Supplementary Fig. S6). In a multivariate Cox regression analysis including tumor size (mm), nodal status (N0/N+), and tumor grade as covariates and DRFI as clinical endpoint, HRD status was nonsignificant. Merging data from the WGS ChemoEndo patients with 761 independent, non-overlapping ChemoEndo-treated patients with unknown HRD status^27^ stratified by PAM50 LumA status, demonstrated that all subgroups had approximately similar outcomes (Fig. 5i). Substantiating the latter, in a multivariate Cox regression model including age, lymph node status and tumor grade as covariates, only lymph node status was statistically significant for DRFI in the combined ChemoEndo-treated patient cohort (Supplementary Fig. S6). Finally, it should be acknowledged that due to the retrospective nature of our cohort with long-term follow-up, no patient received adjuvant therapy including CDK4/6 inhibitors, platinum-based agents, or PARP inhibitors due to national treatment guidelines at the time.

## Discussion

In this study, we show that HRD tumors occur in ERpHER2n BC, though at a lower frequency than in TNBC. Adjusting for population frequencies of different BC subtypes, we provide the estimate that roughly 1 in 20 ERpHER2n BC and 1 in 9 of all BC are HRD. While definitive studies of HRD frequency in population-representative HER2-positive disease and LumA tumors are warranted, these are unlikely to drastically shift overall estimates suggested in this study, as shown in Fig. 1b (frequency estimates with 20% error spans). Notably, while the SCAN-B study in general has been shown to be population representative^25,27^, it is representative of a specific demographic context of patients (Western Europe/Nordics). Consequently, it should be acknowledged that HRD patterns in ERpHER2n patients may differ in other ethnic contexts. Exemplifying the latter, Feng et al. reported an overall HRD frequency of 34.7% in a smaller study based on Chinese BC patients of unclear population representativity, and nearly three times higher HRD frequency in LumA and LumB patients^23^.

From an operational point, this work highlights how global WGS, transcriptomics, and methylation studies can be performed on BC samples collected through routine diagnostic settings across district hospitals in Sweden since 2010 and stored in RNAlater. While successfully achieved previously on 254 TNBC samples^9^, this larger study reinforces that snap frozen tissue collections are not necessary for WGS and/or other global multiomic investigations. The current study also provides an overview of the agreement of both DNA and RNA-based HRD classification methods in ERpHER2n BC, illustrating the challenges faced using different approaches when applied to tumor specimens collected in a routine diagnostic setting without specific tumor tissue enrichment, such as macrodissection.

Crucially, ERpHER2n HRD tumors occur in all PAM50 subtypes, albeit at different frequencies, and do not present a unique transcriptional or DNA methylation profile, especially when acknowledging underlying molecular subtypes (a concept not typically employed in previous studies). Notably, this is in agreement with both the TS228 classification results, showing mixing of HRDetect HRD and HR-proficient tumors in the TS228 HRD group, and with findings in TNBC, where HRD is not distinctively associated with either proposed TNBC mRNA subtypes^60^ nor DNA methylation epitypes (see refs. ^9,45^) Thus, while a PAM50 Basal subtype would indicate a higher probability for HRD, and a LumA subtype the opposite, PAM50 subtypes would still not be particularly useful for selecting ERpHER2n patients for HRD analysis. As the current study lacks complementary gene expression risk score classifications by clinically approved gene expression-based assays, the classification overlap between WGS-based HRD status and mRNA-based risk prediction remains to be investigated. ERpHER2n HRD tumors have general but not universal transcriptional characteristics, including enrichment of proliferation markers and immunogenic potential: while in need of in situ confirmation, HRD ERpHER2n PAM50 Basal and LumB tumors appear more immune infiltrated, and thus possibly more immunogenic than their HR-proficient counterparts. A notable observation in this study was the high HRD frequency (37.5%) in the small subgroup of ERpHER2n tumors subtyped as PAM50 Basal. In these HRD tumors, *BRCA1* gene inactivation occurred in 83.4%, with epigenetic silencing of *BRCA1* by promoter hypermethylation as a dominating feature, similar to TNBC^9^. In contrast, a broader spectrum of inactivated HR genes was observed in LumB tumors, including *RAD51C* and *BRCA1* promoter hypermethylation, but not *BRCA2* and *PALB2* hypermethylation. In HRD ERpHER2n LumB tumors, the *BRCA2*/*PALB2*/*RAD51C* genetic phenotype (see ref. ^9^) constitutes 48.1% of tumors, representing a notable difference to TNBC. Still, the key genetic drivers of HRD in ERpHER2n disease appear similar to TNBC^9^, i.e., *BRCA1*, *BRCA2*, *PALB2*, and *RAD51C*. Of all HRD cases in this study, epigenetic inactivation accounted for approximately 33% of cases, while somatic alterations accounted for approximately 30% additional cases. In comparison, in TNBC, corresponding proportions were approximately 44% and 7%, respectively^9^. Together, our observations support epigenetic silencing as a major HRD cause in both ERpHER2n BC and TNBC^9^. Overall, in this study, a putative inactivation mechanism could be assigned to 71.4% of HRD-positive tumors, representing a conservative lower estimate given the restricted availability of germline status for breast cancer susceptibility genes in our cohort. The repeated observation of approximately 30% of HRD tumors, as determined by HRDetect in this study and in TNBC^9^, without an apparent inactivation mechanism warrants further investigation.

A key objective of this study was to investigate prognostic associations of HRD with conventional standard-of-care (SOC) treatment groups. In our WGS-analyzed Endo cohort, the small collection of HRD tumors stood out as a potential poor outcome subgroup, consistent with their generally lower steroid response and higher tumor proliferation scores compared to HR-proficient tumors. However, the skewing of the Endo cohort towards a more aggressive phenotype emphasizes the need for additional studies assessing the prognostic relevance of HRD in Endo-only patients, given the limitations of the current cohort. Still, these analyses suggest that patients with HRD ERpHER2n tumors may be at risk of poor outcomes if left on Endo treatment alone and should be considered for additional systemic therapy. Consistent with the trend-like association between HRD and poorer outcome in our Endo cohort, a similar observation was reported by Black et al. in a general ERpHER2n patient cohort not stratified for adjuvant therapy^39^. In contrast, population-representative ChemoEndo-treated patients with HRD tumors in our study did not show different DRFI compared to patients with HR-proficient tumors, nor indeed to other stratification statuses, including PAM50 LumA. This key, negative finding, opposite to our TNBC study^9^, stresses the need to continue the search for biomarkers that can stratify ERpHER2n ChemoEndo patients into refined outcome groups to further tailor adjuvant therapies and treatment (de)escalation options. As SCAN-B is not a randomized clinical trial but a representative view of real-world oncology practice, it should be stressed that patient selection for adjuvant therapy (Endo or ChemoEndo) and other specific treatment regimens is not necessarily the same today as during the 2010–2014 inclusion period. For instance, due to national treatment guidelines at the time, no patient received adjuvant therapy including CDK4/6 inhibitors or PARP-inhibitors, requiring additional studies to determine the actual prognostic relevance of HRD (irrespective of inactivation mechanism) in these treatment contexts. Still, while our patients are not treated according to the latest recommended guidelines, it should be acknowledged that such molecularly profiled SOC cohorts with long-term follow-up are currently not available. While there is a lack of consensus regarding how best to manage HRD ERpHER2n BCs, arguments in favor of considering alternative systemic therapies are two-fold. First, HRD has been associated with more favorable patient outcomes after adjuvant chemotherapy in primary TNBC^9^. Second, while in need of substantiation, the small GeparOLA study within early HER2-negative HRD BC reported that patients’ hormone receptor-positive HRD tumors exhibited higher response rates to a combination treatment including olaparib^61,62^. Whether ERpHER2n *BRCA1*/*BRCA2* germline or HRD patients will respond to CDK4/6 inhibitors or antibody-drug conjugates remains unclear, as clinical trial data are scarce^63^. Regardless, while this study raises the hypothesis that patients diagnosed with ERpHER2n HRD tumors may benefit from adjuvant ChemoEndo compared to patients diagnosed with HR-proficient tumors, this needs to be proven in randomized clinical trials.

Our demonstration of increased immune response signatures in HRD ERpHER2n tumors based on RNA-sequencing data is interesting, and in line with previous observations^22^, given ongoing clinical trials involving checkpoint inhibitors in patients with ERpHER2n disease. While RNA-sequencing immune patterns should be confirmed in situ, by for example TIL estimation, and further explored in situ for differences in specific immune cell types by multiplexed IHC or spatial transcriptomics, we recently demonstrated in TNBC that the specific RNA-sequencing immune metagene correlated well with whole-slide H&E pathology estimated TIL counts^59^. Immune checkpoint inhibitors are currently being explored for hormone receptor-positive HER2-negative primary breast cancer in, for example, the KEYNOTE-756 and CheckMate 7FL trials^64,65^. The CheckMate 7FL trial demonstrated that adding the anti-PD-1 agent nivolumab to neoadjuvant chemotherapy significantly improved pathological complete response rates in high-risk ERpHER2n breast cancer, particularly in tumors with high stromal TIL levels and/or PD-L1 positivity. Moreover, the investigators observed a greater benefit in patients with low ER-expressing tumors (<10%), where these features of immunogenicity have previously been shown to be elevated^64,66^. Although ERpHER2n HRD cases in this study were defined by an ER expression ≥10%, our study indicates increased *PD-L1* mRNA expression and immune activation by mRNA signatures in ERpHER2n HRD patients, suggesting that immunotherapy could be of potential clinical benefit to this small patient subset. Finally, in the GIADA trial, it was reported that the combination of a basal-like intrinsic subtype (herein shown to display a high HRD frequency) and high TILs could predict pathological complete response after neoadjuvant treatment with chemotherapy, immune checkpoint inhibition, and endocrine therapy in premenopausal women with aggressive hormone receptor-positive and HER2-negative breast cancer^67^. While several of these features match the characteristics of HRD tumors, it remains to be fully determined whether HRD tumors would benefit from this therapeutic scenario. Taken together, our study highlights the HRD phenotype in ERpHER2n tumors as a marker of aggressiveness, even when compared to HR-proficient tumors within the same molecular subtype, and an interesting association with elevated immune response that should be pursued in more depth. We demonstrate that genomic HRD status  provides additional information beyond intrinsic molecular BC subtypes in categorizing tumors and understanding which patients might need further treatment. Moreover, this study shows that HRD is much less frequent in ERpHER2n tumors compared to TNBC, suggesting that the overall HRD frequency in a Nordic/Western European demographic context is likely between 10-13%, but that the same key drivers of DNA repair deficiency are identified in both breast cancer groups. Beyond prediction of HRD status, WGS has the potential to offer comprehensive genomic reporting beyond HRD for each patient. In the recent study by Black et al.^39^, analyzing a population-based breast cancer cohort of approximately 2500 patients, it was reported that WGS could identify potential markers of precision medicine in approximately 27% of patients. These markers may serve as triage tools for the prediction of response to targeted therapies, identify potential treatment resistance, and inform about recruitment potential for prospective clinical trials. If further substantiated in relevant SOC settings, this may argue for the future clinical value of WGS in a general breast cancer context, in which HRD prediction may represent one of several read-outs.

## Supplementary information

Supplementary Information

Description of Additional Supplementary files

Supplementary Data 1

Supplementary Data 2

Supplementary Data 3

Supplementary Data 4

Supplementary Data 5

## Data Availability

The raw whole genome sequencing data for SCAN-B cases are protected and currently not available due to data privacy laws and specific patient consent. The processed somatic whole genome sequence tumor data (i.e., called somatic variants) can be made available for academic use only upon reasonable request, depending on the request’s alignment with Swedish data privacy laws, ethical permissions, and specific informed patient consent, defined through a formal data request application. Data requests should be made to the SCAN-B Steering Group, using the SCAN-B research project application template form and contact address [scanb@med.lu.se] listed on the SCAN-B website [https://www.scan-b.lu.se/en/scientists]. Processing time of initial requests is estimated at 6–8 weeks depending on the scheduled steering group meetings. Depending on the nature of a request and the geographic location of the applicant/host university, additional data transfer agreements may be required as determined by data protection officers at Lund University, Sweden, to assure that any relevant and current legal restrictions imposed by Swedish law and the European Union concerning research data sharing are followed. The DNA methylation data used in this study were reported by Hohmann et al.^38^ and is deposited in the Gene Expression Omnibus database under accession code GSE278586. The SCAN-B RNA-sequencing data used in this study are available from Staaf et al.^27^ through an online repository [https://data.mendeley.com/datasets/yzxtxn4nmd/3]. Source data are provided with this paper (Supplementary Data 1).
