# Supplementary material for: Homologous recombination deficiency in primary ER-positive and HER2-negative breast cancer
Source: Commun Med (Lond). 2026 Feb 16;6:118. doi: 10.1038/s43856-026-01385-0 (PMC12909304; doi:10.1038/s43856-026-01385-0)
Supplement: Supplementary file 1 — Supplementary Information [file 43856_2026_1385_MOESM1_ESM.pdf]

A)

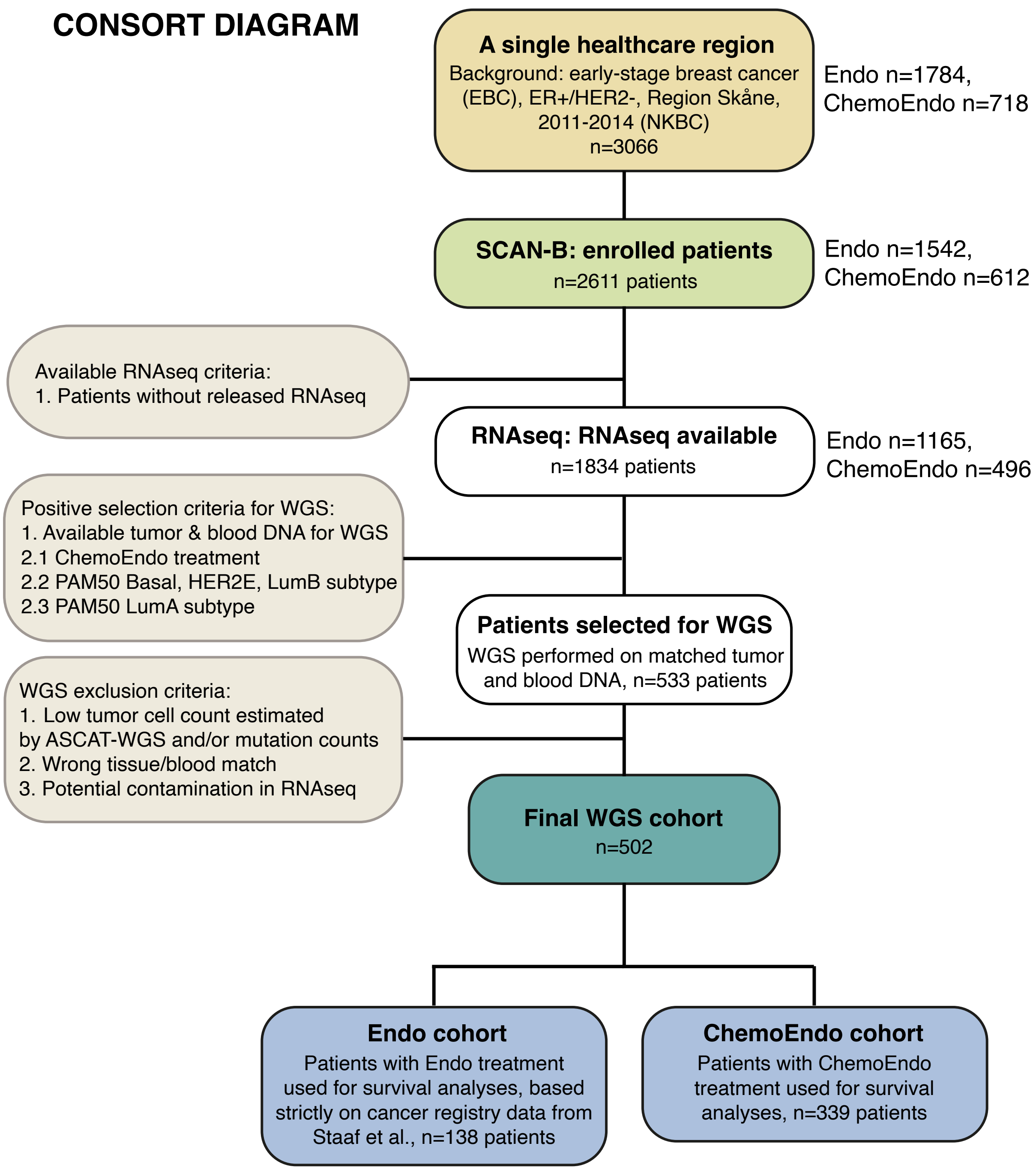

Supplementary Figure S1. CONSORT and treatment group representativity. (A) CONSORT diagram. NKBC: Swedish National Quality Registry for Breast cancer. **(B)** Study overview, with key analyses and external cohorts used. **(C)** Representativity of the WGS profiled Endo treatment group compared to background patient populations diagnosed during 2010-2014 for different clinical variables obtained from the NKBC registry in the catchment region (RS: Skane healthcare region). EBC: Early breast cancer (surgically treated). Top axis in barplots corresponds to sample numbers. **(D)** Representativity of the WGS profiled ChemoEndo treatment group compared to background patient populations for different clinical variables as in panel C. Top axis in barplots corresponds to sample numbers.

B) Study layout

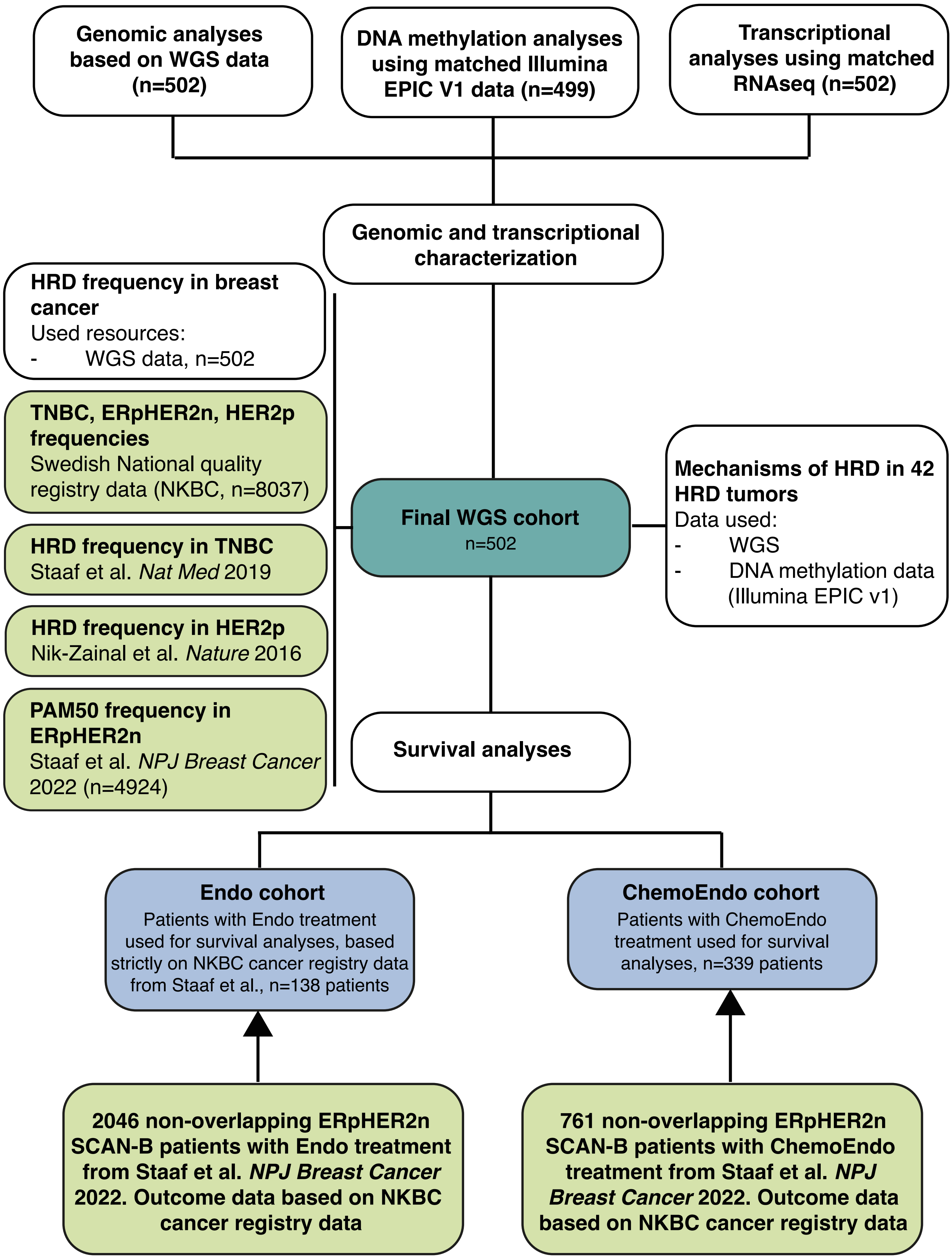

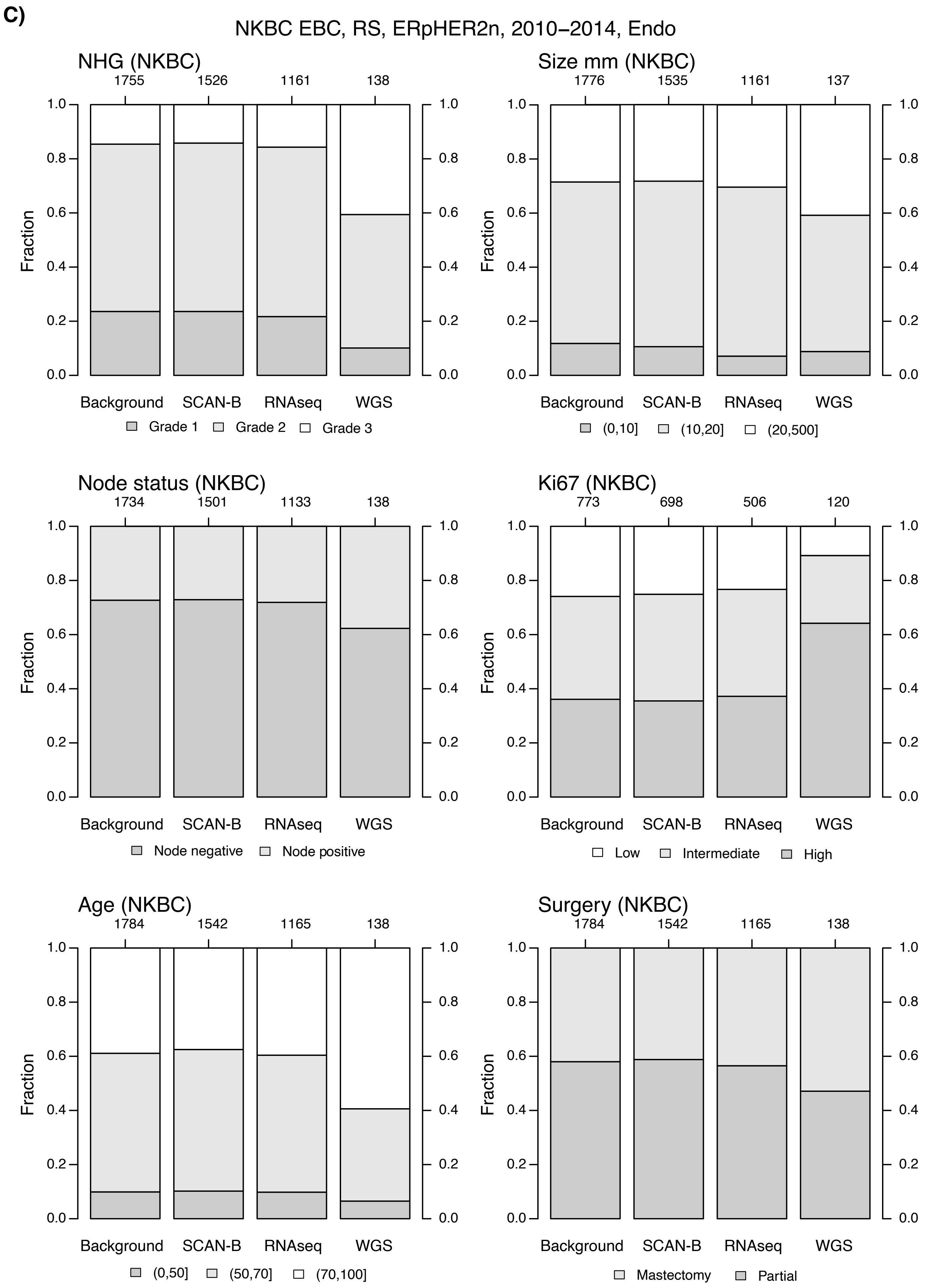
C)
NKBC EBC, RS, ERpHER2n, 2010−2014, Endo
NHG (NKBC)
1755 1526 1161 138
Fraction
Background SCAN-B RNAseq WGS
Grade 1 Grade 2 Grade 3
Size mm (NKBC)
1776 1535 1161 137
(0,10] (10,20] (20,500]
Node status (NKBC)
1734 1501 1133 138
Node negative Node positive
Ki67 (NKBC)
773 698 506 120
Low Intermediate High
Age (NKBC)
1784 1542 1165 138
(0,50] (50,70] (70,100]
Surgery (NKBC)
1784 1542 1165 138
Mastectomy Partial

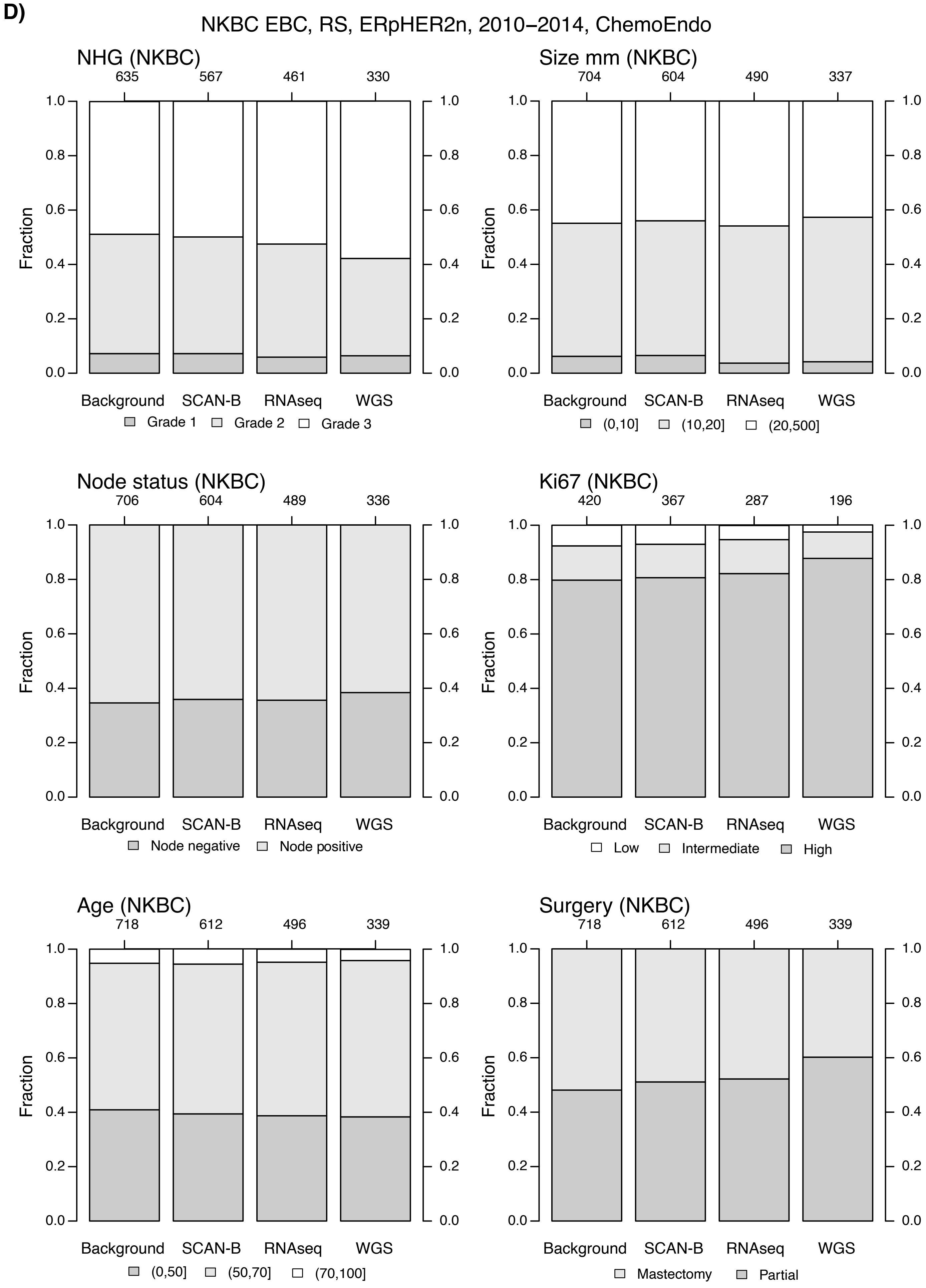
D)
NKBC EBC, RS, ERpHER2n, 2010–2014, ChemoEndo
NHG (NKBC)
635 567 461 330
Fraction
Background SCAN-B RNAseq WGS
Grade 1 Grade 2 Grade 3
Size mm (NKBC)
704 604 490 337
(0,10] (10,20] (20,500]
Node status (NKBC)
706 604 489 336
Node negative Node positive
Ki67 (NKBC)
420 367 287 196
Low Intermediate High
Age (NKBC)
718 612 496 339
(0,50] (50,70] (70,100]
Surgery (NKBC)
718 612 496 339
Mastectomy Partial

A)

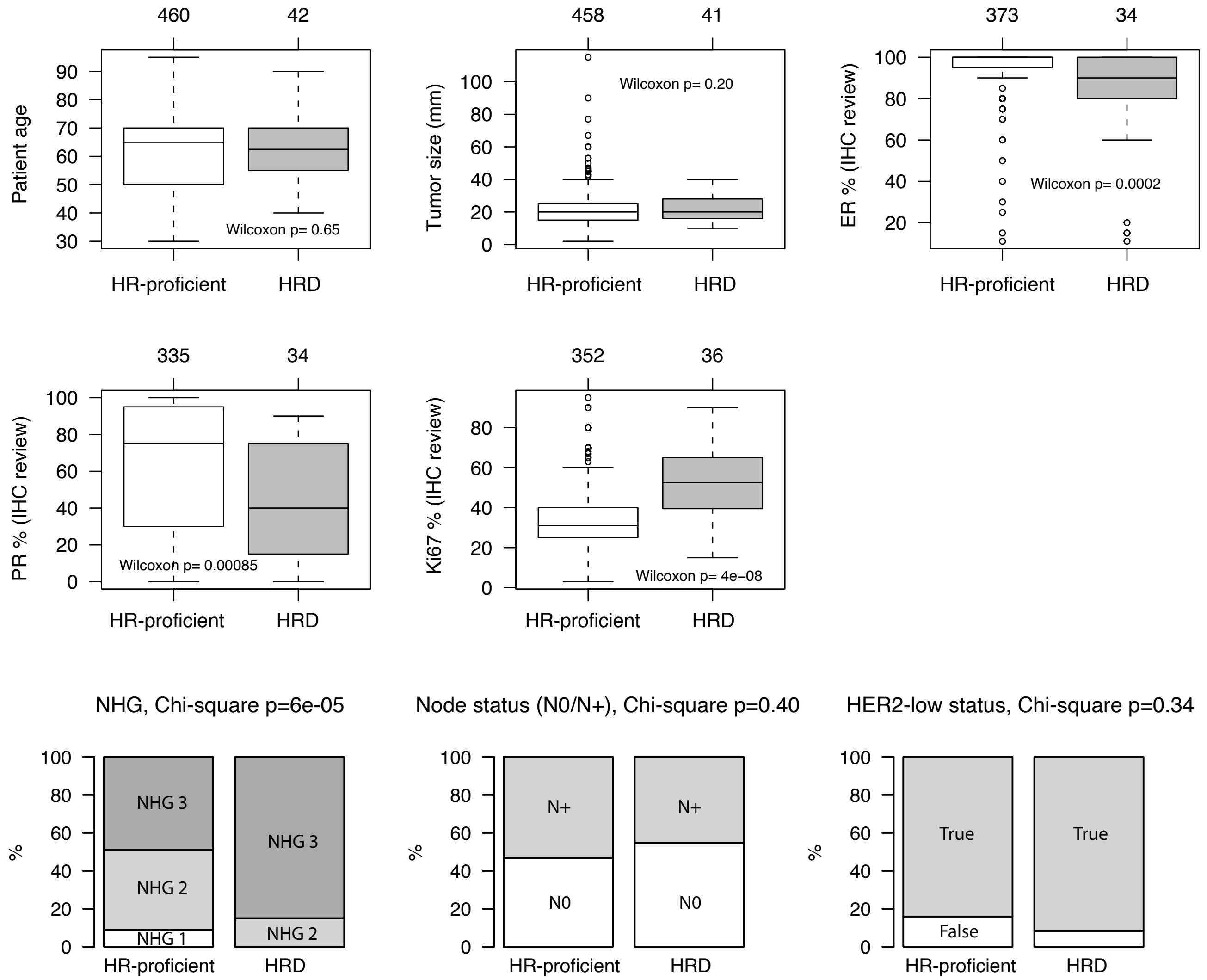

Supplementary Figure S2. Clinicopathological characteristics of HRD and HR-proficient ERpHER2n tumors in 502 WGS analyzed patients divided by treatment status. (A) Characteristics for all 502 patients. Not all patients have review data available. **(B)** Characteristics for 339 ChemoEndo treated patients. **(C)** Characteristics for 138 Endo treated patients. Not all patients have review data available. NHG: Nottingham grade index. N0: lymph node negative. N+: lymph node positive. Boxplot elements correspond to: i) center line = median, ii) box limits = upper and lower quartiles, iii) whiskers = 1.5x interquartile range. In boxplots, top axes indicate group sizes.

B)

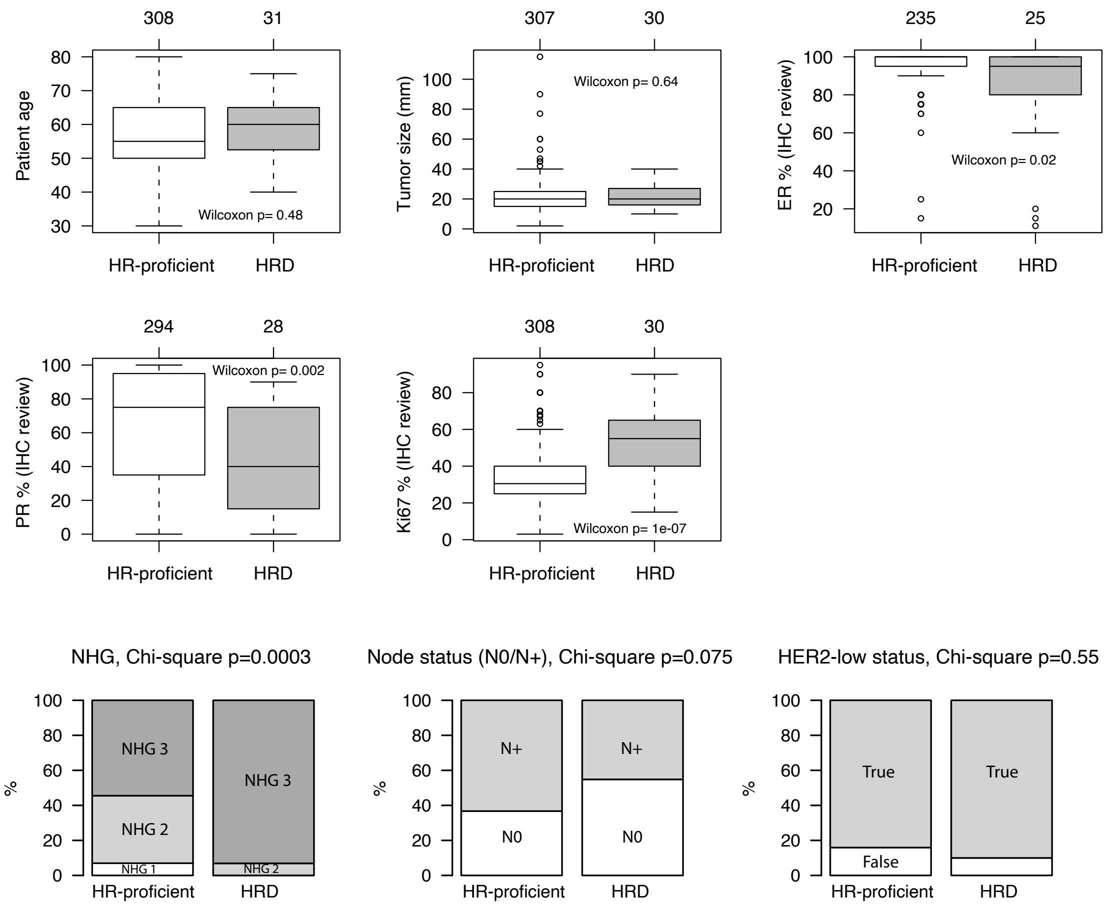
308
31
Patient age
Wilcoxon p= 0.48
HR-proficient
HRD
307
30
Tumor size (mm)
Wilcoxon p= 0.64
235
25
ER % (IHC review)
Wilcoxon p= 0.02
294
28
PR % (IHC review)
Wilcoxon p= 0.002
308
30
Ki67 % (IHC review)
Wilcoxon p= 1e-07
NHG, Chi-square p=0.0003
NHG 3
NHG 2
NHG 1
Node status (N0/N+), Chi-square p=0.075
N+
N0
HER2-low status, Chi-square p=0.55
True
False
%

C)

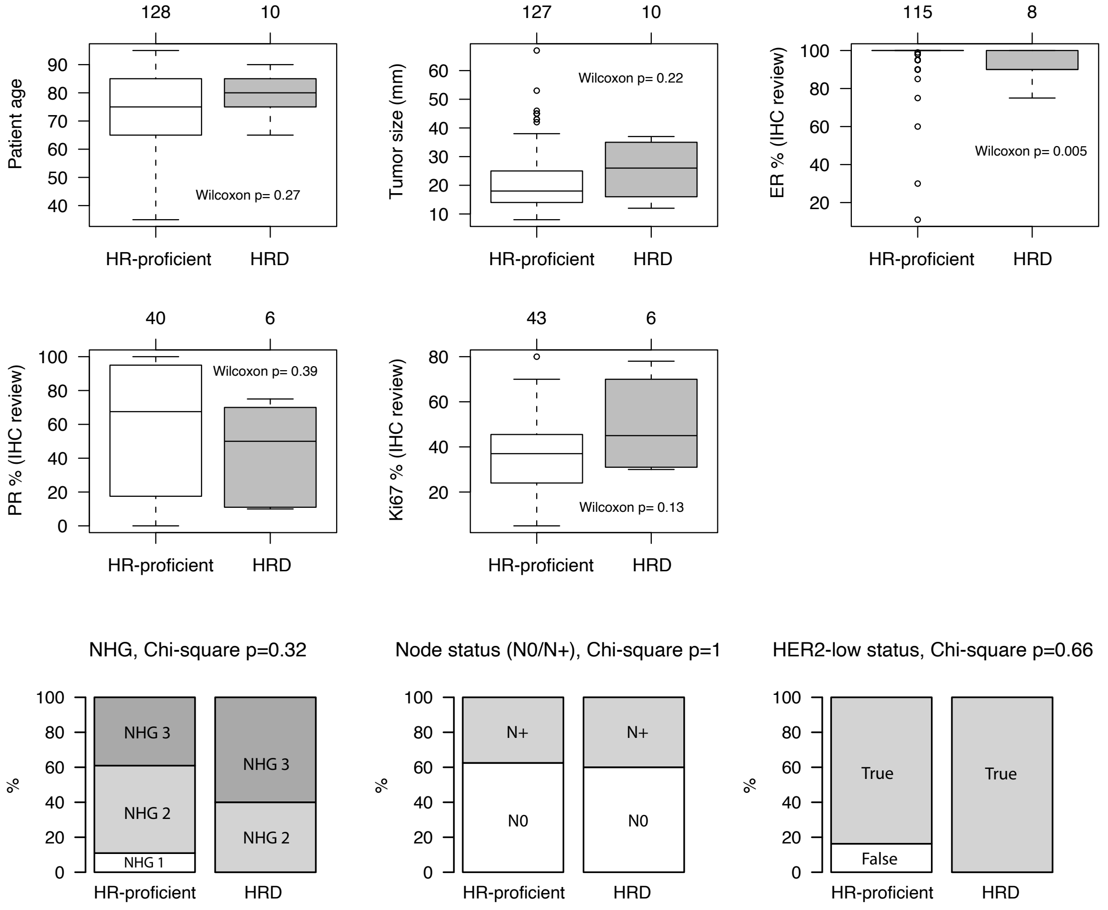
128
10
Patient age
Wilcoxon p= 0.27
HR-proficient
HRD
127
10
Tumor size (mm)
Wilcoxon p= 0.22
115
8
ER % (IHC review)
Wilcoxon p= 0.005
40
6
PR % (IHC review)
Wilcoxon p= 0.39
43
6
Ki67 % (IHC review)
Wilcoxon p= 0.13
NHG, Chi-square p=0.32
NHG 3
NHG 2
NHG 1
Node status (N0/N+), Chi-square p=1
N+
N0
HER2-low status, Chi-square p=0.66
True
False
%

A) PAM50 Basal

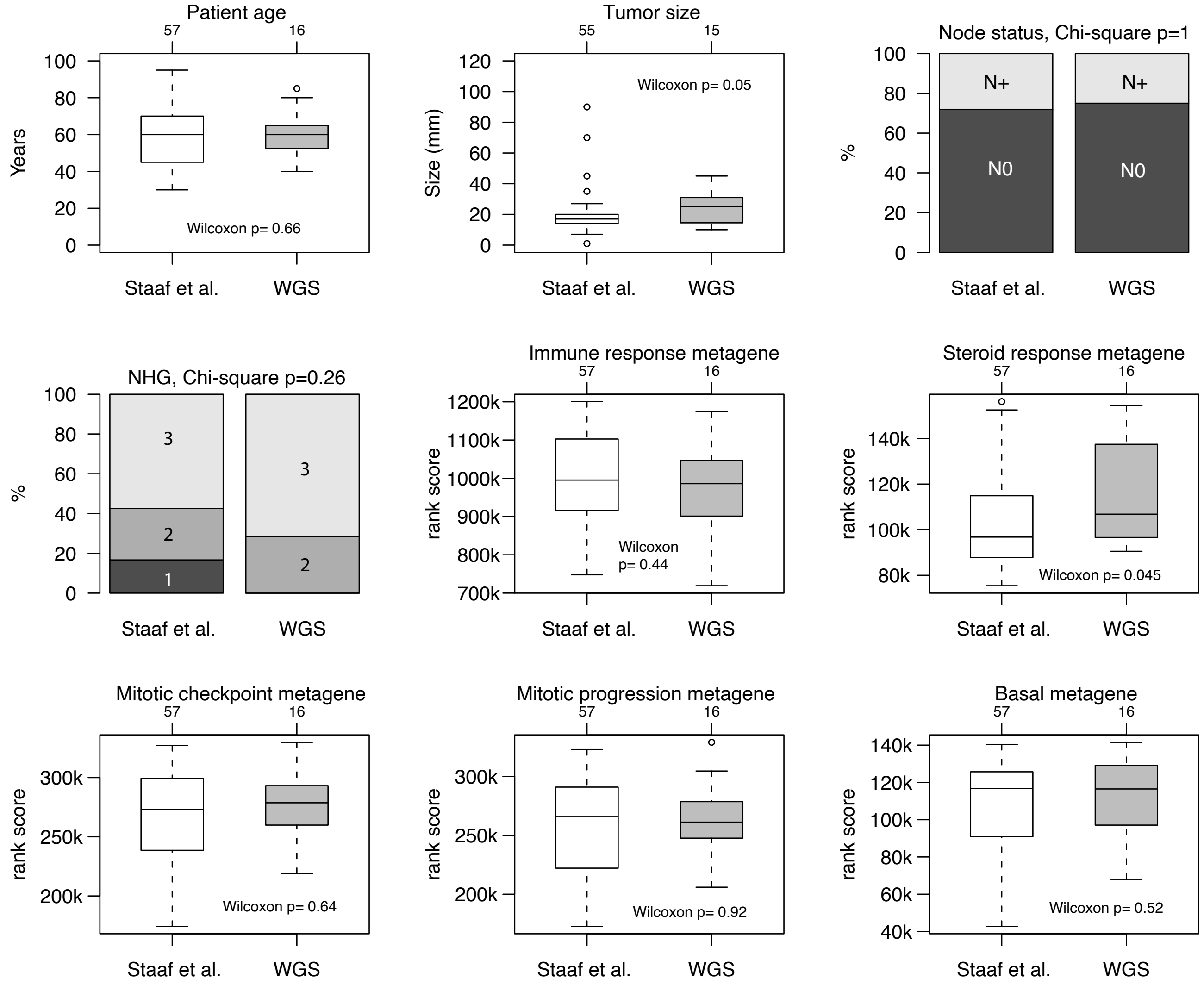

Supplementary Figure S3. Representativity of PAM50 subtypes in the ERpHER2n WGS cohort versus PAM50 subtypes in 4427 unrelated ERpHER2n tumors from the study by Staaf et al. (NPJ Breast Cancer 2022) regarding clinicopathological characteristics and expression of biological mRNA metagenes from Fredlund et al. (BCR 2012). The Chi-square test was used to test statistical significance for categorical data, whereas Wilcoxon's test was used for continuous data and rank-scores. (**A**) PAM50 Basal. (**B**) PAM50 HER2E. (**C**) PAM50 LumA. (**D**) PAM50 LumB. (**E**) PAM50 Normal. While certain characteristics in panel D (LumB) are significant it should be noted that the larger group sizes here can cause smaller differences to become statistically significant (e.g. age and nodal status). NHG: Nottingham grade index (grade 1, 2, 3). N0: lymph node negative. N+: lymph node positive.

B) PAM50 HER2E

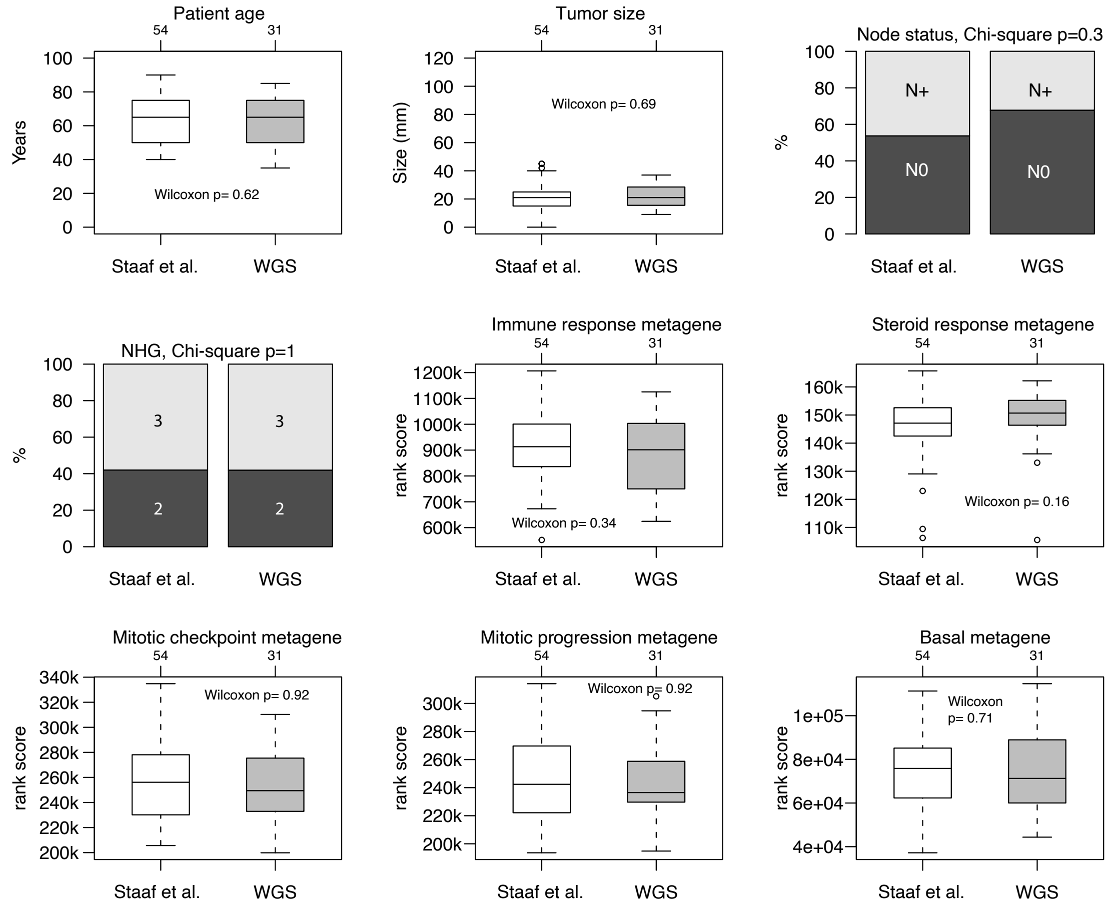

C) PAM50 LumA

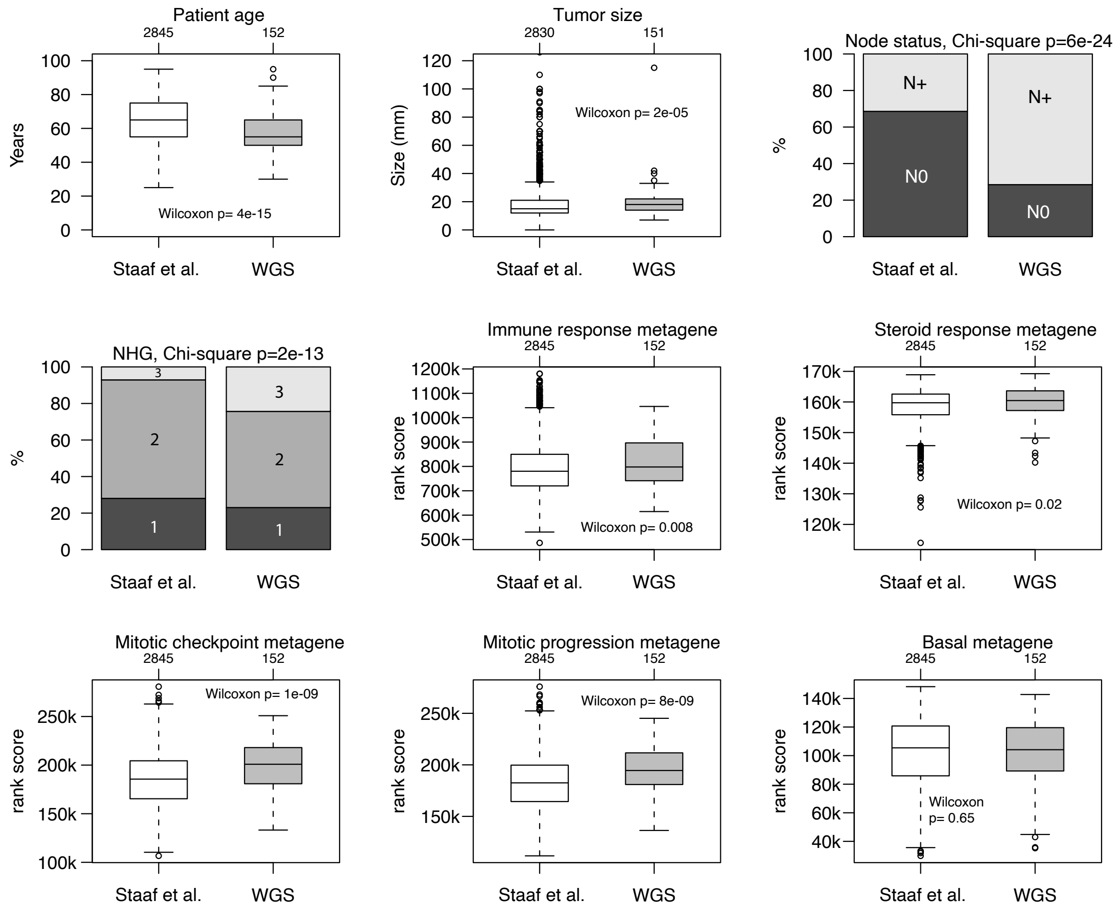

D) PAM50 LumB

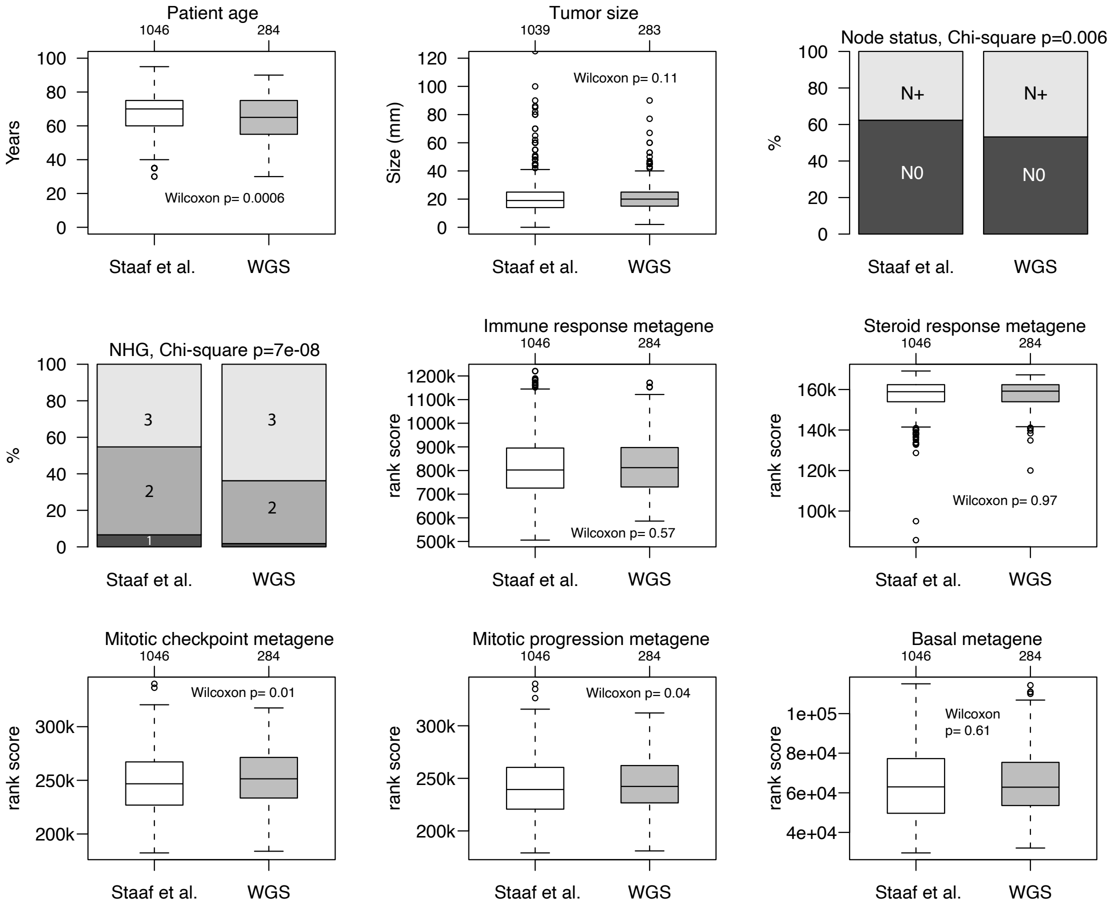

E) PAM50 Normal

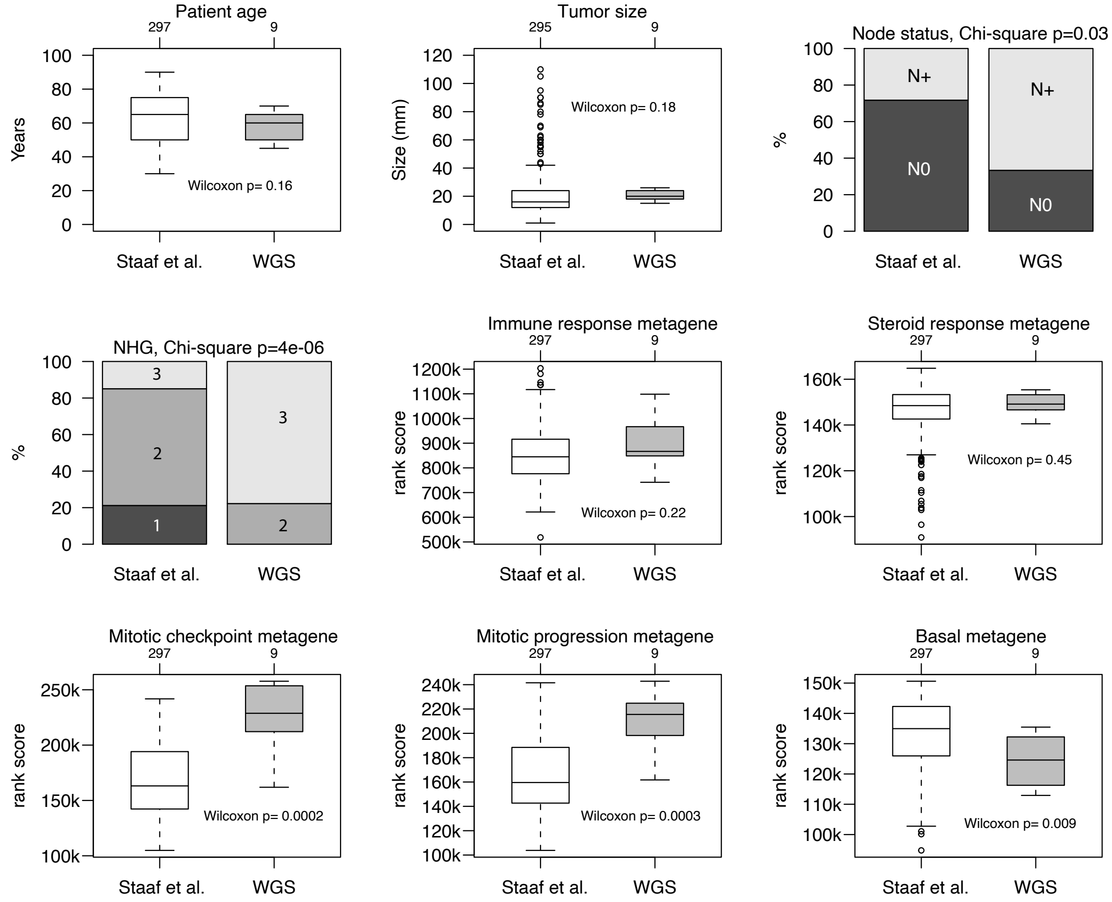

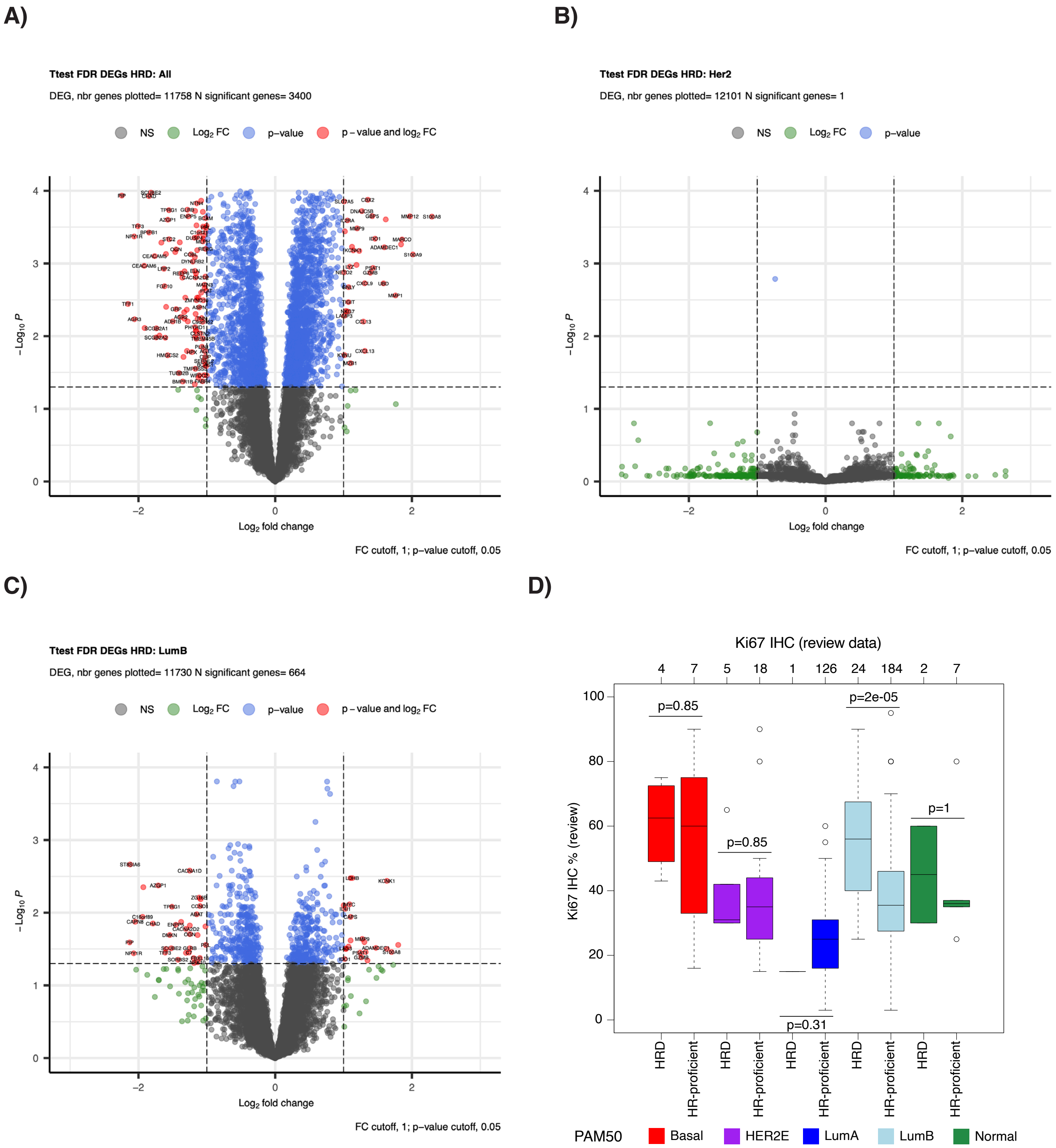

Supplementary Figure S4. Gene expression analysis of ERpHER2n tumors with respect to HRD status. (**A**) Volcano plot showing the results of the differential gene expression analysis for HRD-status in all 502 tumors. (**B**) Volcano plot showing the results of the differential gene expression analysis for HRD-status in HER2E tumors. (**C**) Volcano plot showing the results of the differential gene expression analysis for HRD-status in LumB tumors. In A-C, the fold change (FC) cut-off was 1, and the p-value FDR cut-off 0.05. (**D**) Ki67 IHC expression based on clinical review data for tumors stratified by PAM50 and HRD status, showing higher Ki67 levels in HRD LumB tumors. Two-sided p-values calculated using Wilcoxon's test. (**E**) Principal component analysis (PCA) of FPKM data in all ERpHER2n tumors using the top 10000 most varying genes. HRD tumors are marked by black points. Principal component 1 and 2 (PC1 and PC2, respectively) are shown. (**F**) PCA plot of all tumors using the 1000 most variant genes with a minimum FPKM level >1. (**G**) PCA plot of LumB tumors using the 10000 most variant genes. (**H**) PCA plot of LumB tumors using the 1000 most variant genes with a minimum FPKM level >1.

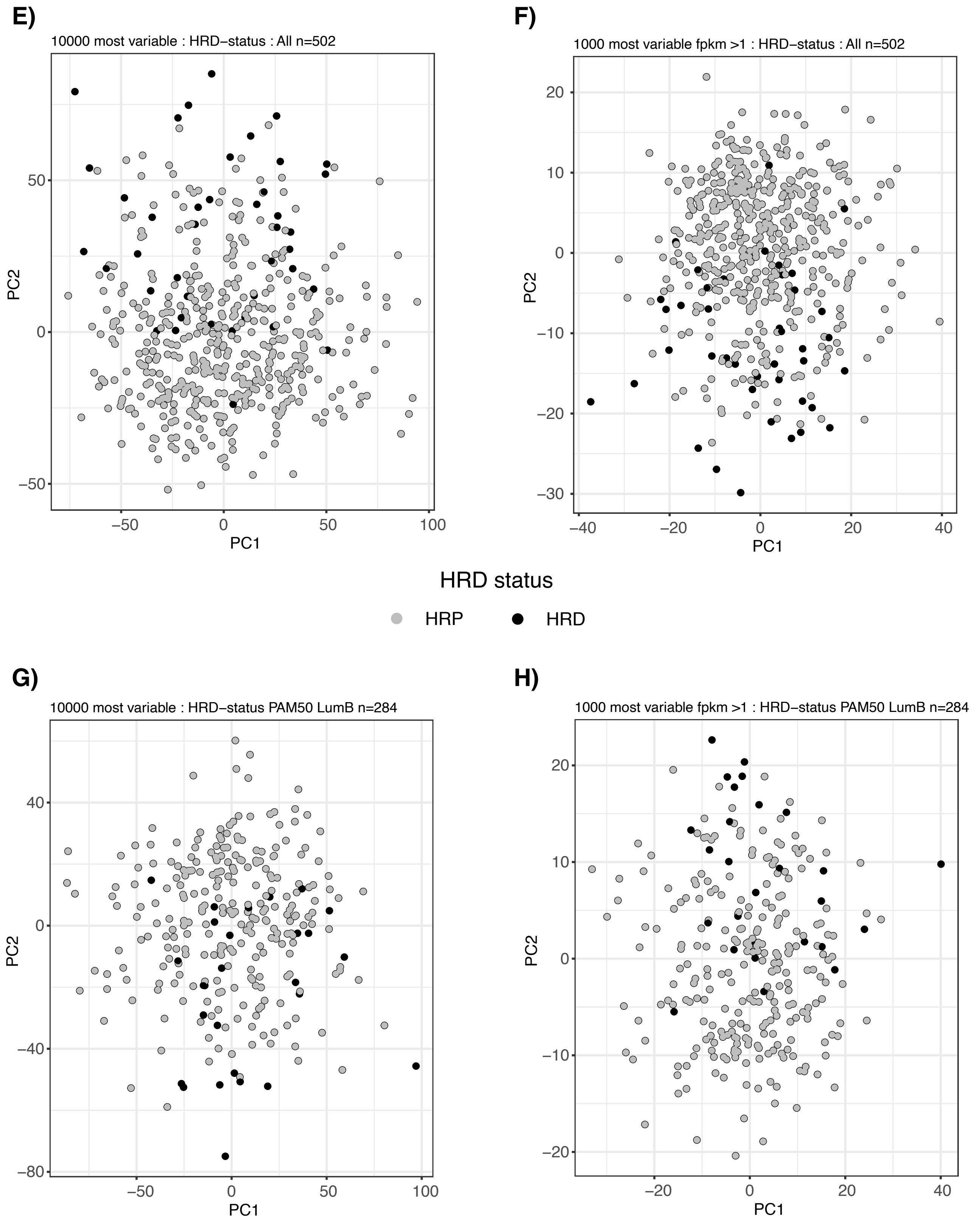

E)
10000 most variable : HRD−status : All n=502
F)
1000 most variable fpkm >1 : HRD−status : All n=502
HRD status
HRP
HRD
G)
10000 most variable : HRD−status PAM50 LumB n=284
H)
1000 most variable fpkm >1 : HRD−status PAM50 LumB n=284
PC1
PC2

A)

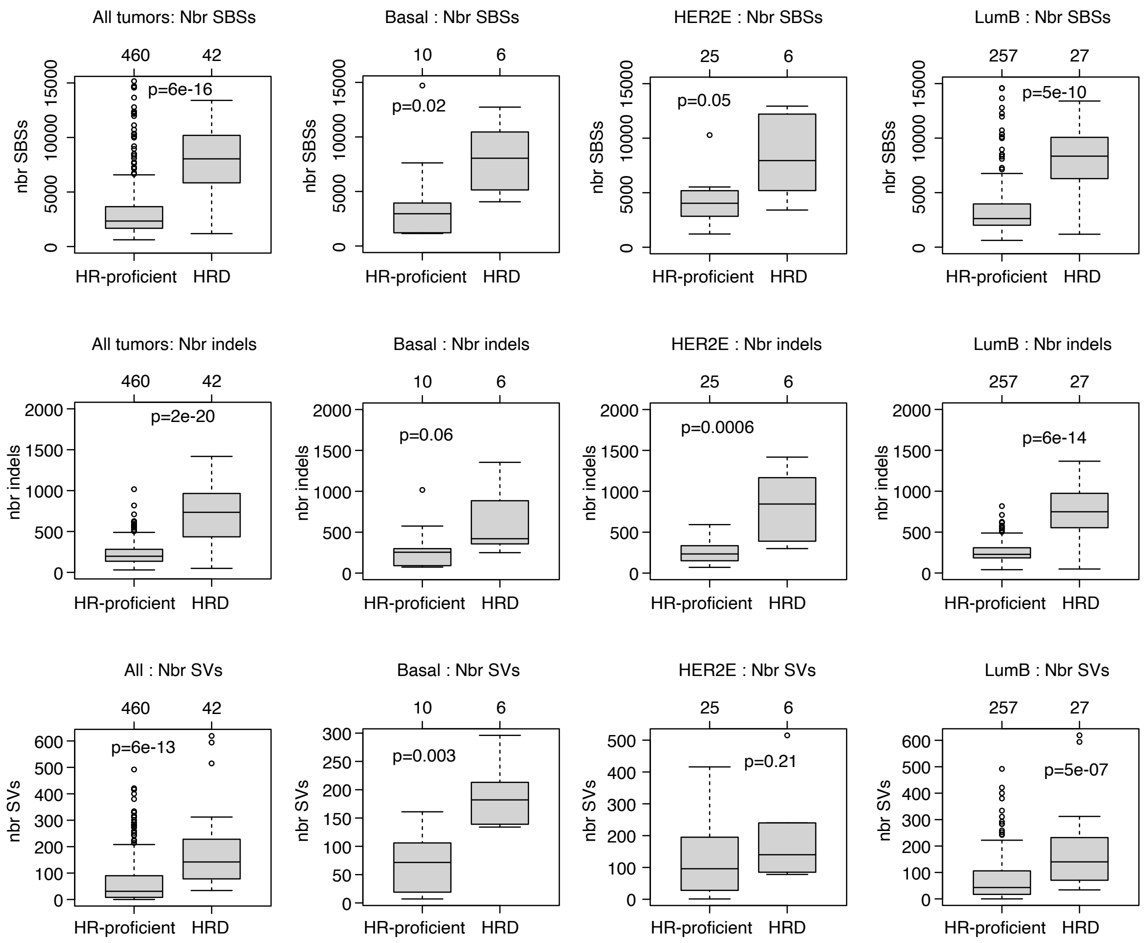

Supplementary Figure S5. Genetic features of HRD ERpHER2n tumors. (A) Number of single base substitutions (SBSs), indels, and structural rearrangements (SVs) in HRD versus HR-proficient tumors stratified by PAM50 subtype (LumA and Normal excluded due to few HRD cases). Two-sided p-values are calculated using Wilcoxon's test. **(B)** Exposure to SBS signatures for all tumors, Basal, HER2E, and LumB. Only SBS1, SBS3, SBS5, SBS8, and SBS2/13 are shown as these were the signatures with significant differences between groups. Two-sided p-values are calculated using Wilcoxon's test. **(C)** Exposure to SV signatures for all tumors, Basal, HER2E, and LumB subtypes. Only signatures significant or borderline nonsignificant are shown. Two-sided p-values are calculated using Wilcoxon's test. **(D)** Exposure to indel signatures for all tumors, Basal, HER2E, and LumB subtypes. Only signatures with significant differences are shown. Two-sided p-values are calculated using Wilcoxon's test. **(E)** WGS estimated tumor ploidy, fraction of the genome altered by copy number alterations (CN_FGA), fraction of the genome altered by LOH (LOH_FGA), and number of ASCAT segments on chromosome 1-22 per tumor called as gain or loss for all tumors, Basal, HER2E, and LumB subtypes. **(F)** Barplot of FDR adjusted Wilcoxon's test p-values (-log10 transformed) for 25 copy number signatures (CN1-25) between HR-proficient and HRD tumors stratified by PAM50 subtype. E.g. for CN12, significant p-values (FDR>0.05) was found for the comparison of HRD vs HR-proficient tumors in the total cohort and in LumB tumors specifically. **(G)** Left: frequency of gene driver events based on somatic SBSs and indels in PAM50 Basal tumors stratified by HRD status. Right: frequency of gene driver events based on somatic structural rearrangements (SVs) in PAM50 Basal tumors stratified by HRD status. Only genes detected in at least two tumors in at least one group are shown. **(H)** As in G but for PAM50 HER2E tumors. (**I**) Principal component analysis (PCA) of tumor purity adjusted DNA methylation data (beta values) for three different CpG contexts in all tumors and PAM50 subtypes (LumA excluded as only one tumor was HRD). For each context, the 5000 most variant CpGs in each subgroup of samples were selected for PCA. Black dots represent tumors with an HRDetect HRD classification. First two principal components (PC1 and PC2) shown. (**J**) PCA of tumor purity adjusted DNA methylation data (beta values) for three different CpG contexts in LumB HRD tumors. For each context, the 5000 most variant CpGs in the subgroup were selected for PCA. Samples are colored based on their proposed HRD inactivation mechanism. First two principal components (PC1 and PC2) shown.

B)

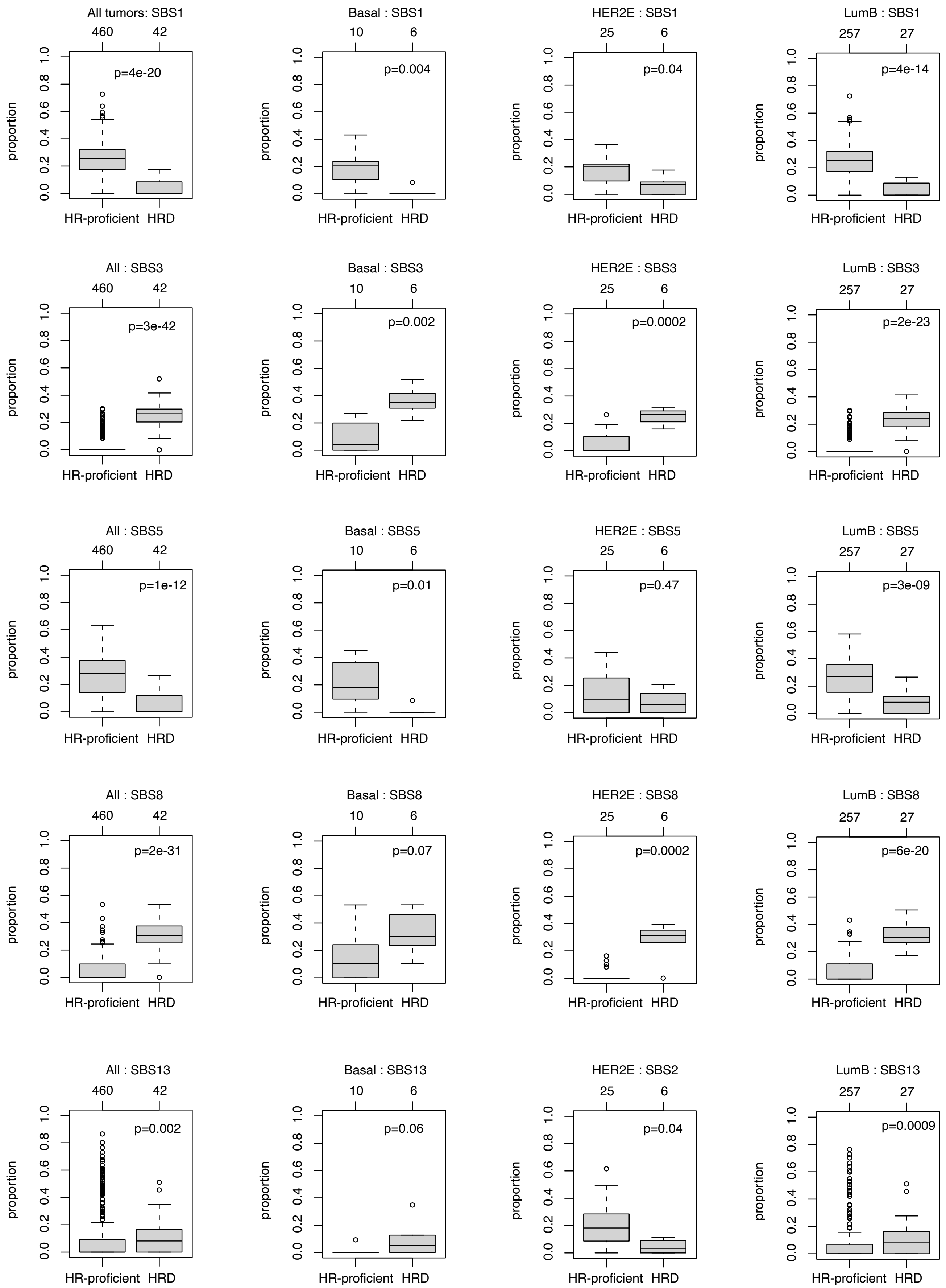
All tumors: SBS1
460 42
p=4e-20
Basal : SBS1
10 6
p=0.004
HER2E : SBS1
25 6
p=0.04
LumB : SBS1
257 27
p=4e-14
All : SBS3
460 42
p=3e-42
Basal : SBS3
10 6
p=0.002
HER2E : SBS3
25 6
p=0.0002
LumB : SBS3
257 27
p=2e-23
All : SBS5
460 42
p=1e-12
Basal : SBS5
10 6
p=0.01
HER2E : SBS5
25 6
p=0.47
LumB : SBS5
257 27
p=3e-09
All : SBS8
460 42
p=2e-31
Basal : SBS8
10 6
p=0.07
HER2E : SBS8
25 6
p=0.0002
LumB : SBS8
257 27
p=6e-20
All : SBS13
460 42
p=0.002
Basal : SBS13
10 6
p=0.06
HER2E : SBS2
25 6
p=0.04
LumB : SBS13
257 27
p=0.0009
proportion
0.0 0.2 0.4 0.6 0.8 1.0
HR-proficient HRD

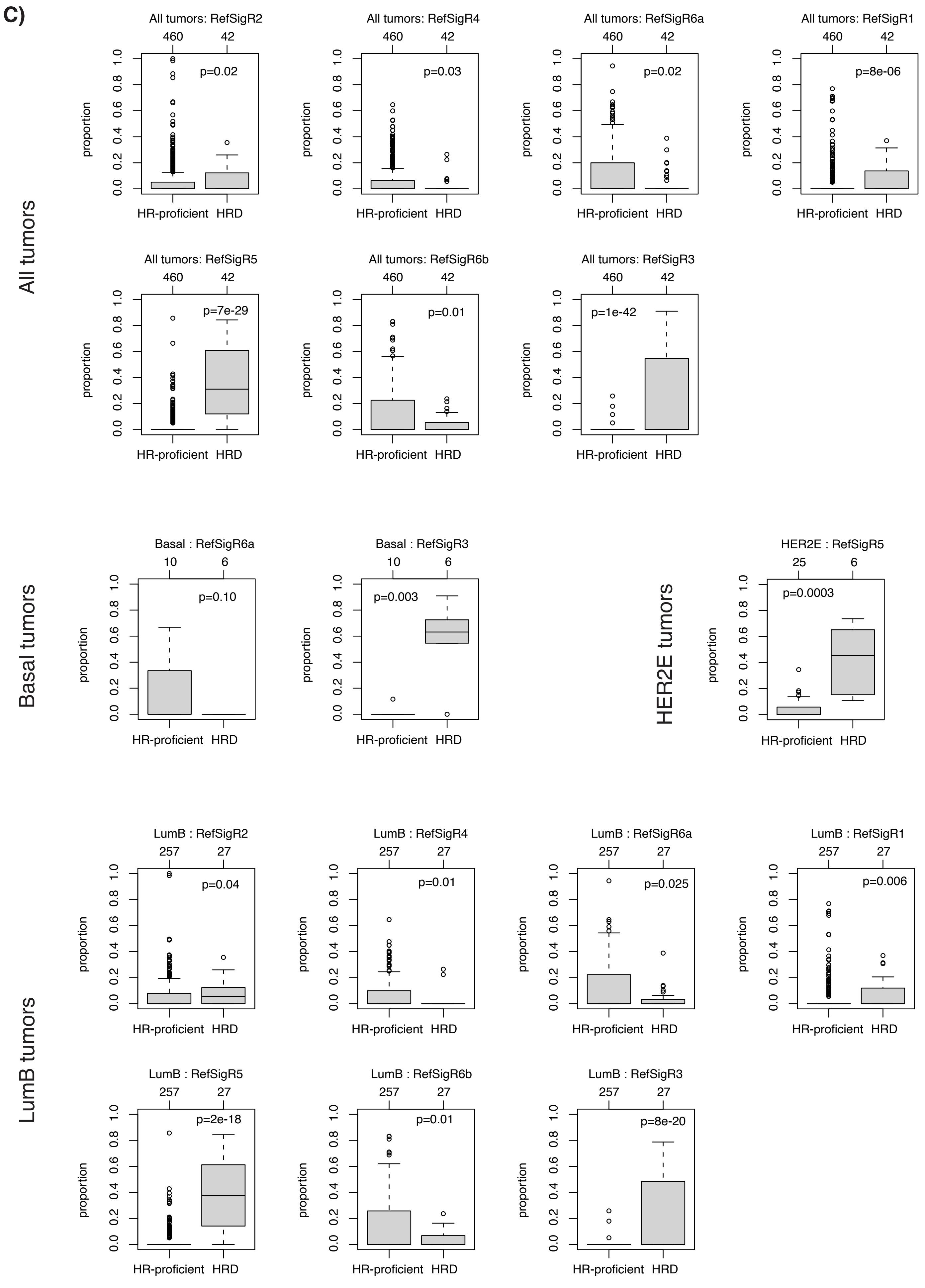
C)
All tumors
All tumors: RefSigR2
460 42
p=0.02
All tumors: RefSigR4
460 42
p=0.03
All tumors: RefSigR6a
460 42
p=0.02
All tumors: RefSigR1
460 42
p=8e-06
All tumors: RefSigR5
460 42
p=7e-29
All tumors: RefSigR6b
460 42
p=0.01
All tumors: RefSigR3
460 42
p=1e-42
Basal tumors
Basal : RefSigR6a
10 6
p=0.10
Basal : RefSigR3
10 6
p=0.003
HER2E tumors
HER2E : RefSigR5
25 6
p=0.0003
LumB tumors
LumB : RefSigR2
257 27
p=0.04
LumB : RefSigR4
257 27
p=0.01
LumB : RefSigR6a
257 27
p=0.025
LumB : RefSigR1
257 27
p=0.006
LumB : RefSigR5
257 27
p=2e-18
LumB : RefSigR6b
257 27
p=0.01
LumB : RefSigR3
257 27
p=8e-20
proportion
HR-proficient
HRD

D)

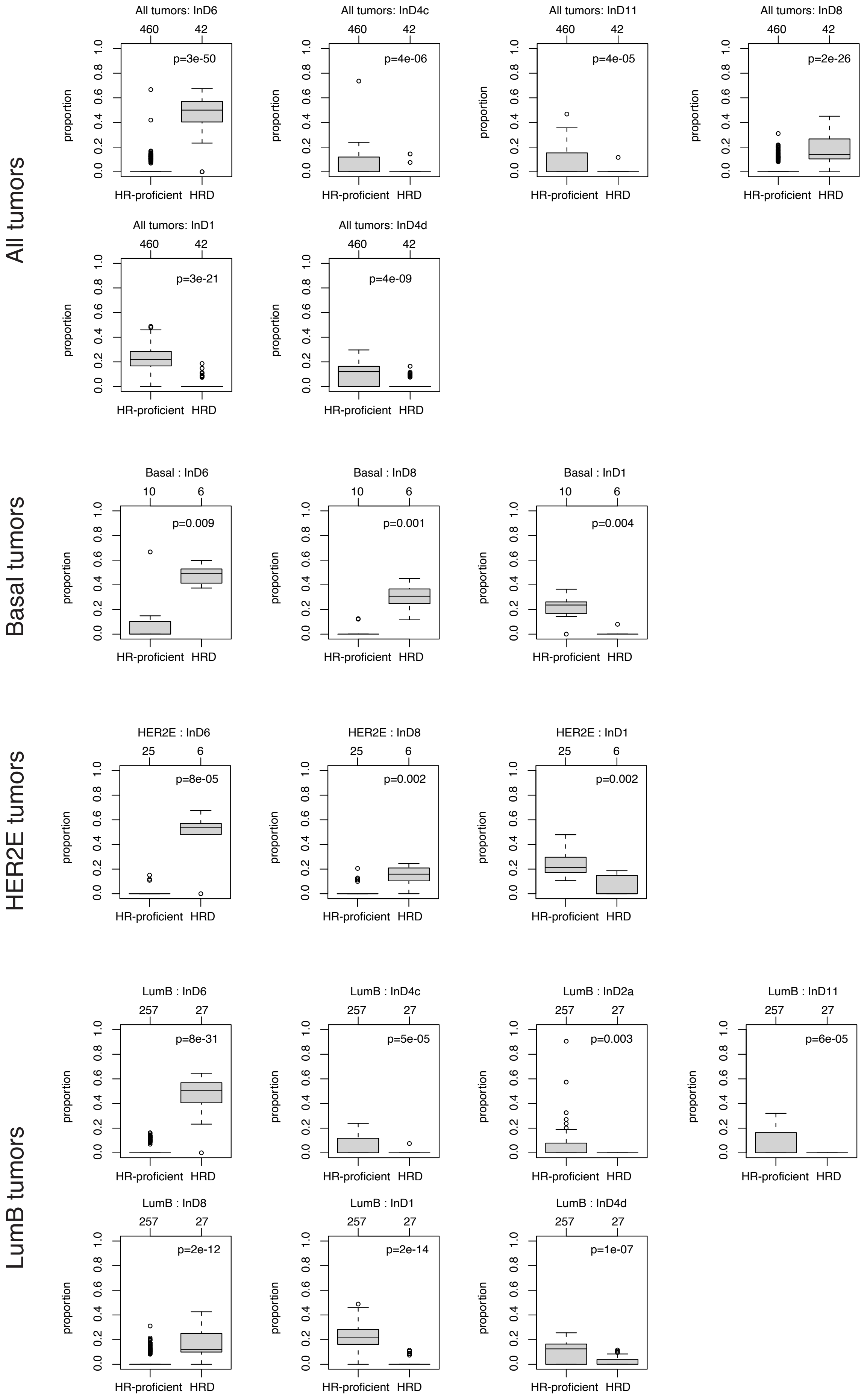

All tumors
All tumors: InD6
460 42
p=3e-50
All tumors: InD4c
460 42
p=4e-06
All tumors: InD11
460 42
p=4e-05
All tumors: InD8
460 42
p=2e-26
All tumors: InD1
460 42
p=3e-21
All tumors: InD4d
460 42
p=4e-09
Basal tumors
Basal : InD6
10 6
p=0.009
Basal : InD8
10 6
p=0.001
Basal : InD1
10 6
p=0.004
HER2E tumors
HER2E : InD6
25 6
p=8e-05
HER2E : InD8
25 6
p=0.002
HER2E : InD1
25 6
p=0.002
LumB tumors
LumB : InD6
257 27
p=8e-31
LumB : InD4c
257 27
p=5e-05
LumB : InD2a
257 27
p=0.003
LumB : InD11
257 27
p=6e-05
LumB : InD8
257 27
p=2e-12
LumB : InD1
257 27
p=2e-14
LumB : InD4d
257 27
p=1e-07
proportion
0.0 0.2 0.4 0.6 0.8 1.0
HR-proficient HRD

E)

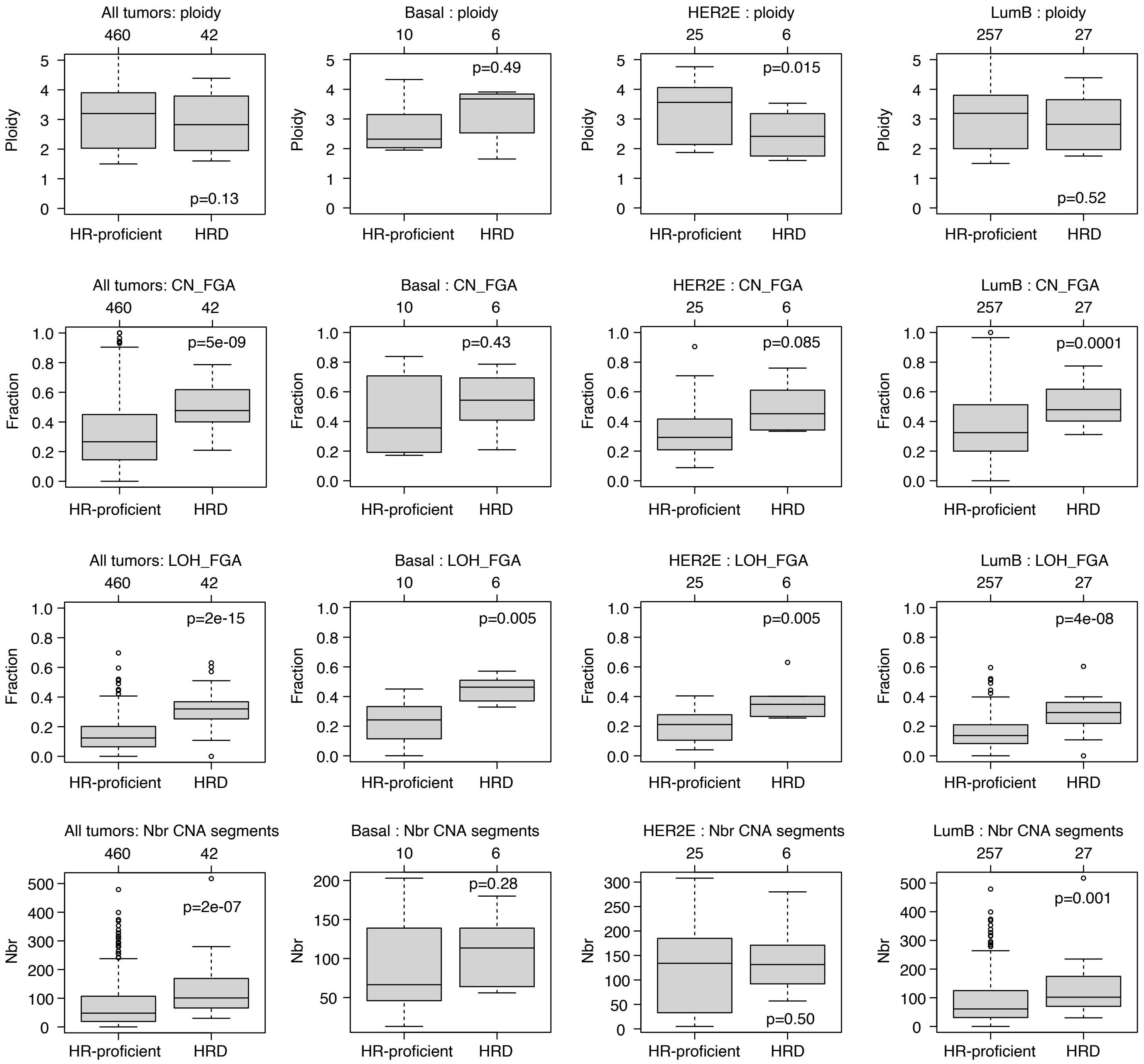
All tumors: ploidy
460 42
Ploidy
p=0.13
HR-proficient HRD
Basal : ploidy
10 6
p=0.49
HER2E : ploidy
25 6
p=0.015
LumB : ploidy
257 27
p=0.52
All tumors: CN_FGA
Fraction
p=5e-09
Basal : CN_FGA
p=0.43
HER2E : CN_FGA
p=0.085
LumB : CN_FGA
p=0.0001
All tumors: LOH_FGA
p=2e-15
Basal : LOH_FGA
p=0.005
HER2E : LOH_FGA
p=0.005
LumB : LOH_FGA
p=4e-08
All tumors: Nbr CNA segments
Nbr
p=2e-07
Basal : Nbr CNA segments
p=0.28
HER2E : Nbr CNA segments
p=0.50
LumB : Nbr CNA segments
p=0.001

F)

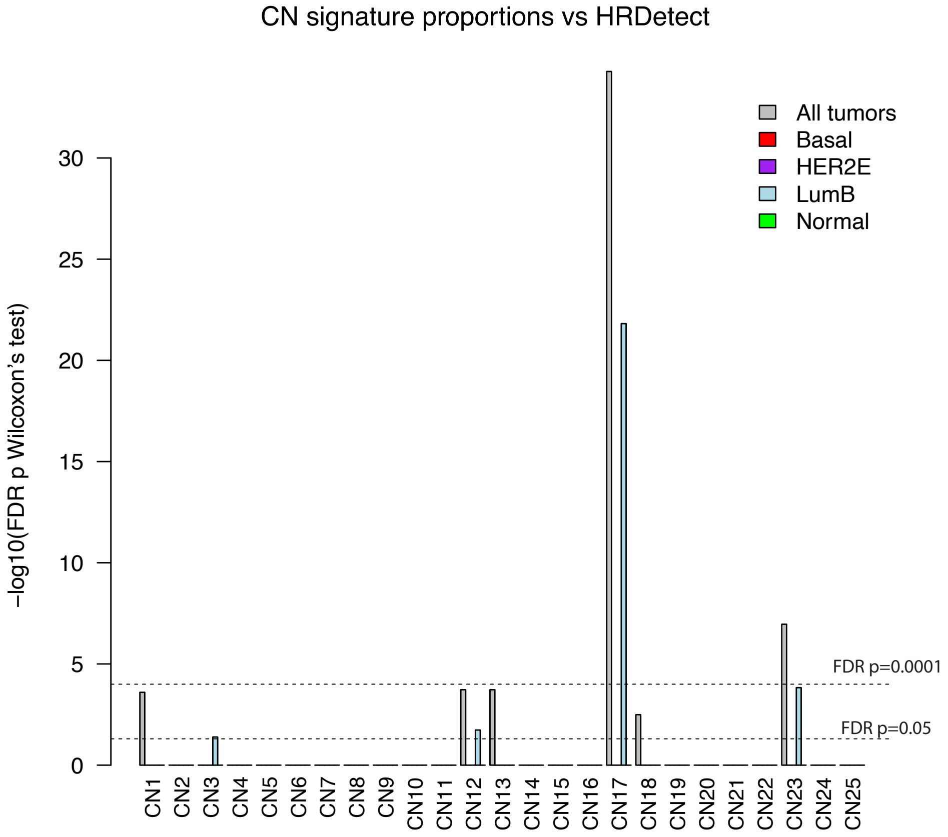

G) PAM50 Basal

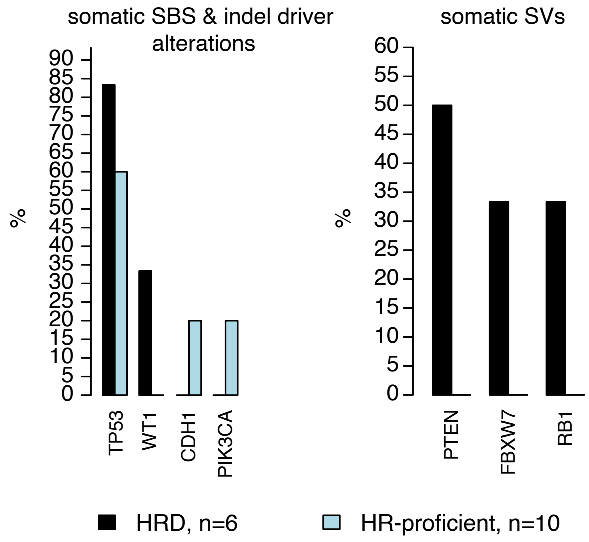

H) PAM50 HER2E

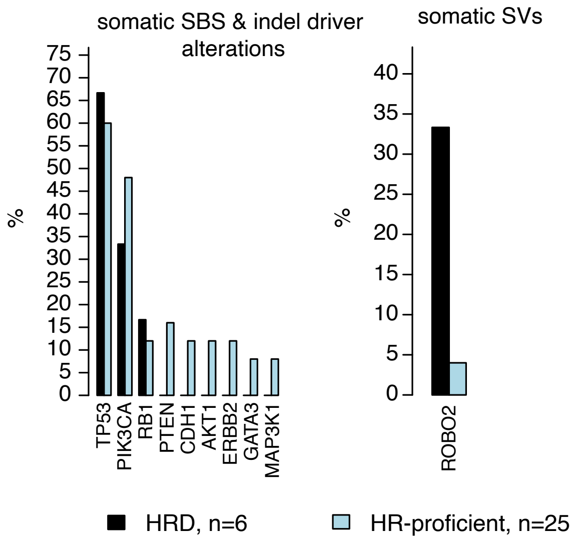

I)

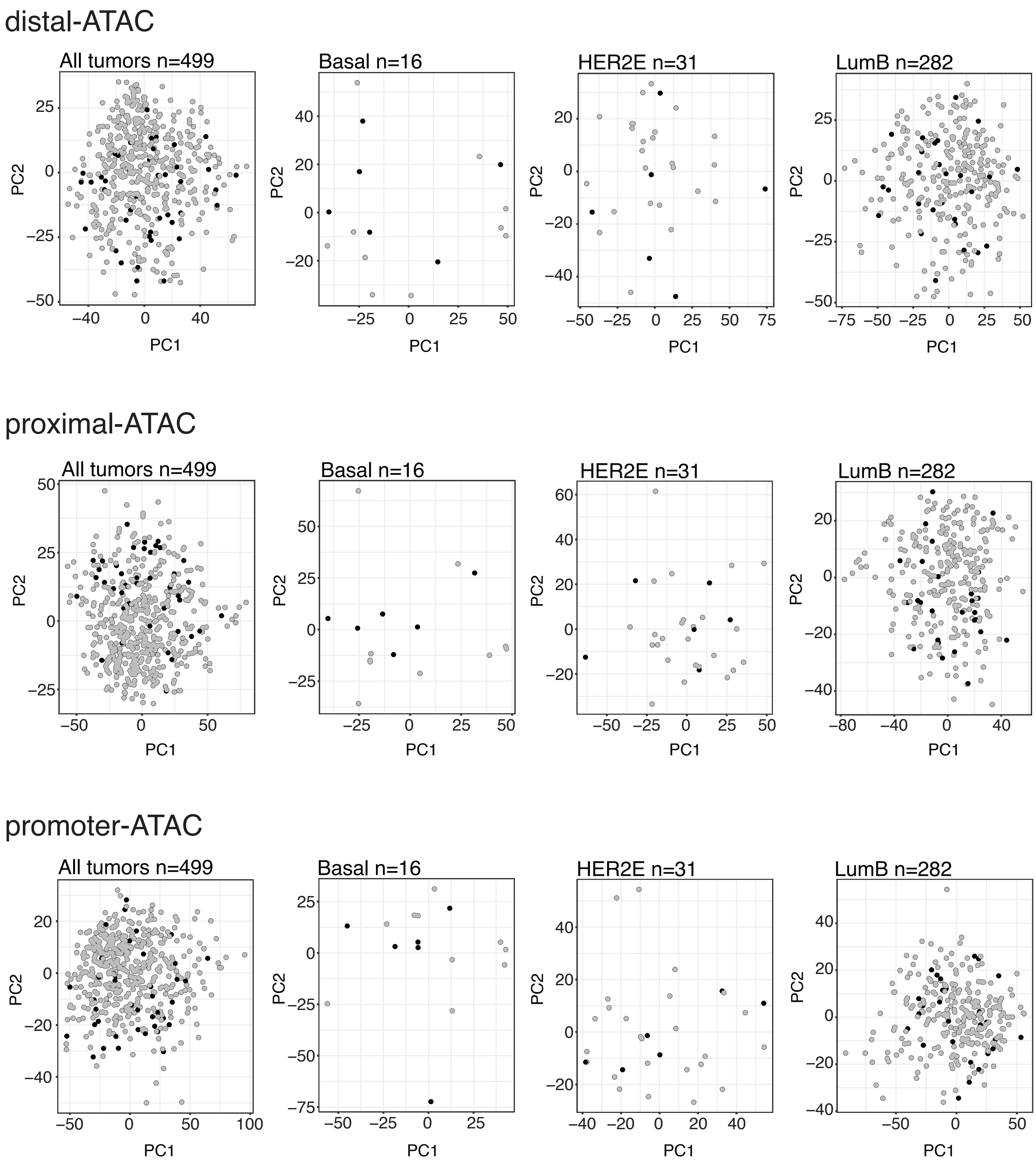

distal-ATAC
All tumors n=499
Basal n=16
HER2E n=31
LumB n=282
proximal-ATAC
All tumors n=499
Basal n=16
HER2E n=31
LumB n=282
promoter-ATAC
All tumors n=499
Basal n=16
HER2E n=31
LumB n=282
PC1
PC2

J)

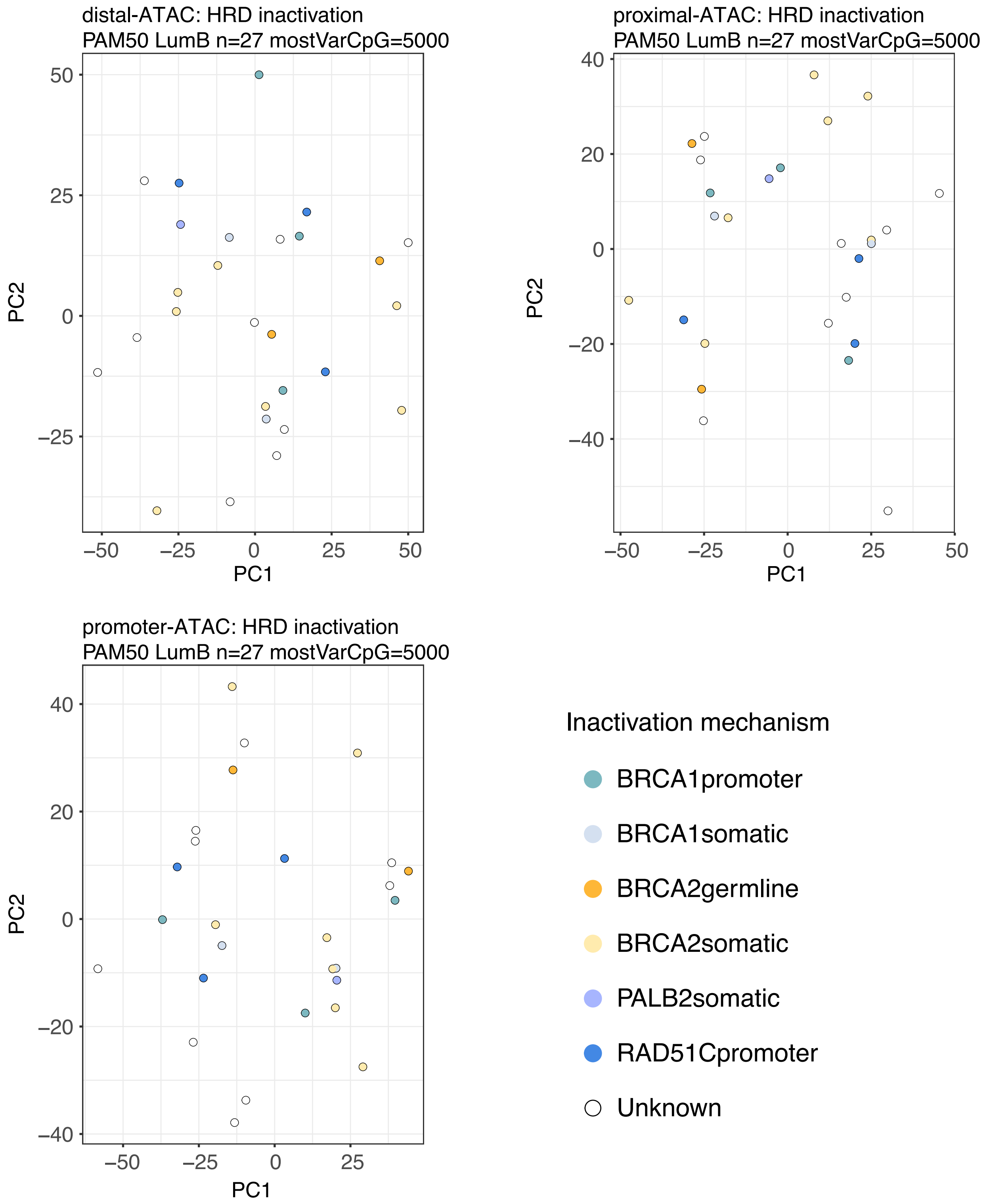
distal-ATAC: HRD inactivation
PAM50 LumB n=27 mostVarCpG=5000
PC1
PC2
proximal-ATAC: HRD inactivation
PAM50 LumB n=27 mostVarCpG=5000
PC1
PC2
promoter-ATAC: HRD inactivation
PAM50 LumB n=27 mostVarCpG=5000
PC1
PC2
Inactivation mechanism
BRCA1promoter
BRCA1somatic
BRCA2germline
BRCA2somatic
PALB2somatic
RAD51Cpromoter
Unknown

A)

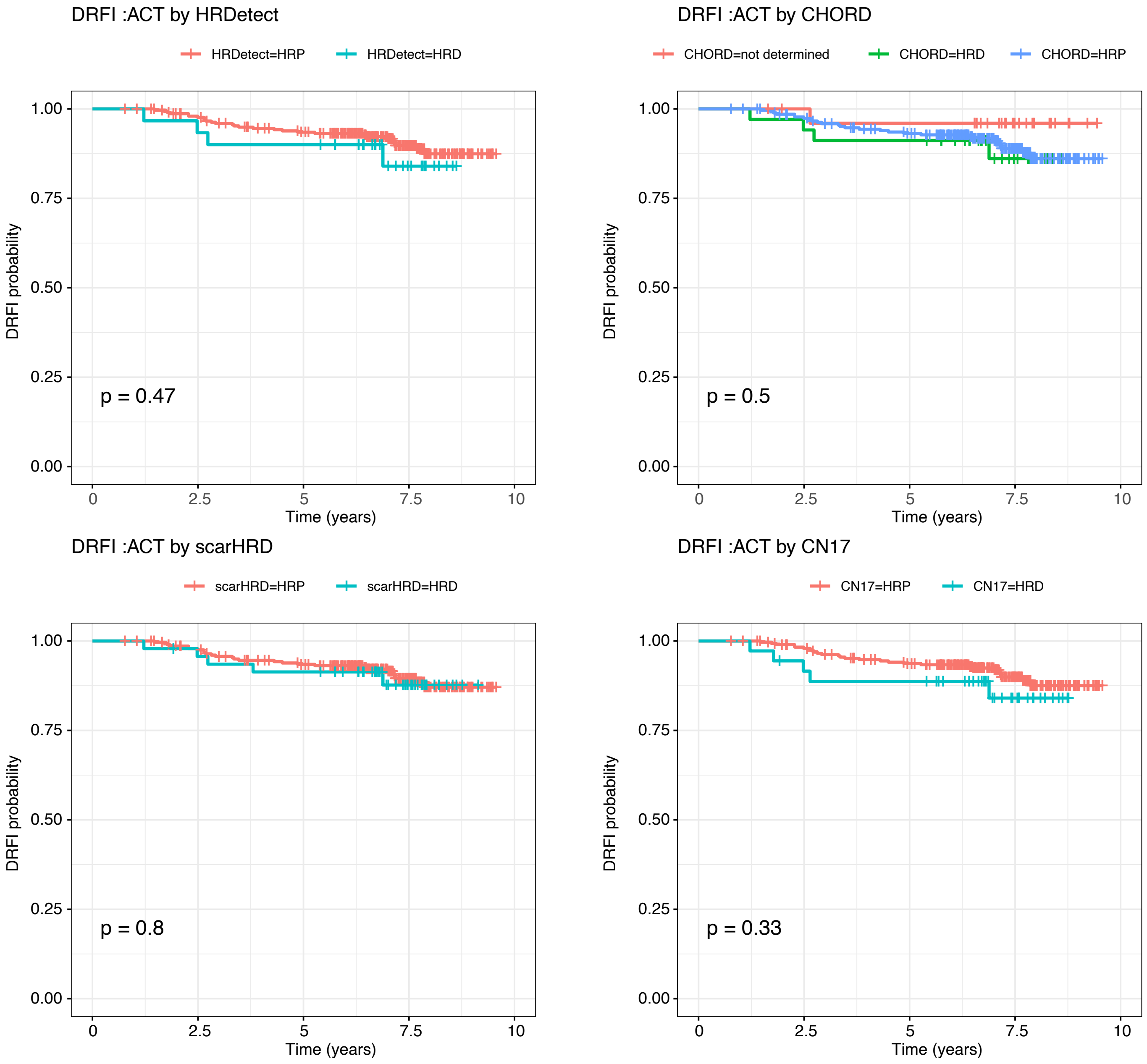

Supplementary Figure S6. Survival analysis of alternative HRD methods and multivariate Cox regression in ChemoEndo ERpHER2n BC. (A) Kaplan-Meier plots of alternative HRD classification methods using distant relapse-free interval (DRFI) as clinical endpoint in ChemoEndo patients. P-values computed using the log-rank test. **(B)** Forest plot of hazard ratios and 95% confidence intervals for a multivariate Cox regression model including a merged molecular class (PAM50 LumA yes/no and HRD status), Nottingham grade index (NHG), binary age, and binary lymph node status in ChemoEndo treated ERpHER2n patients using distant relapse-free interval (DRFI) as clinical endpoint. In this analysis, WGS-analyzed ChemoEndo treated patients was merged with 761 non-overlapping ChemoEndo treated patients with unknown HRD status stratified by their PAM50 LumA status (LumA or not-LumA). For the 761 patients the DRFI data based on cancer registry data from, while for the WGS analyzed samples clinical review DRFI data was used as endpoint.

B)

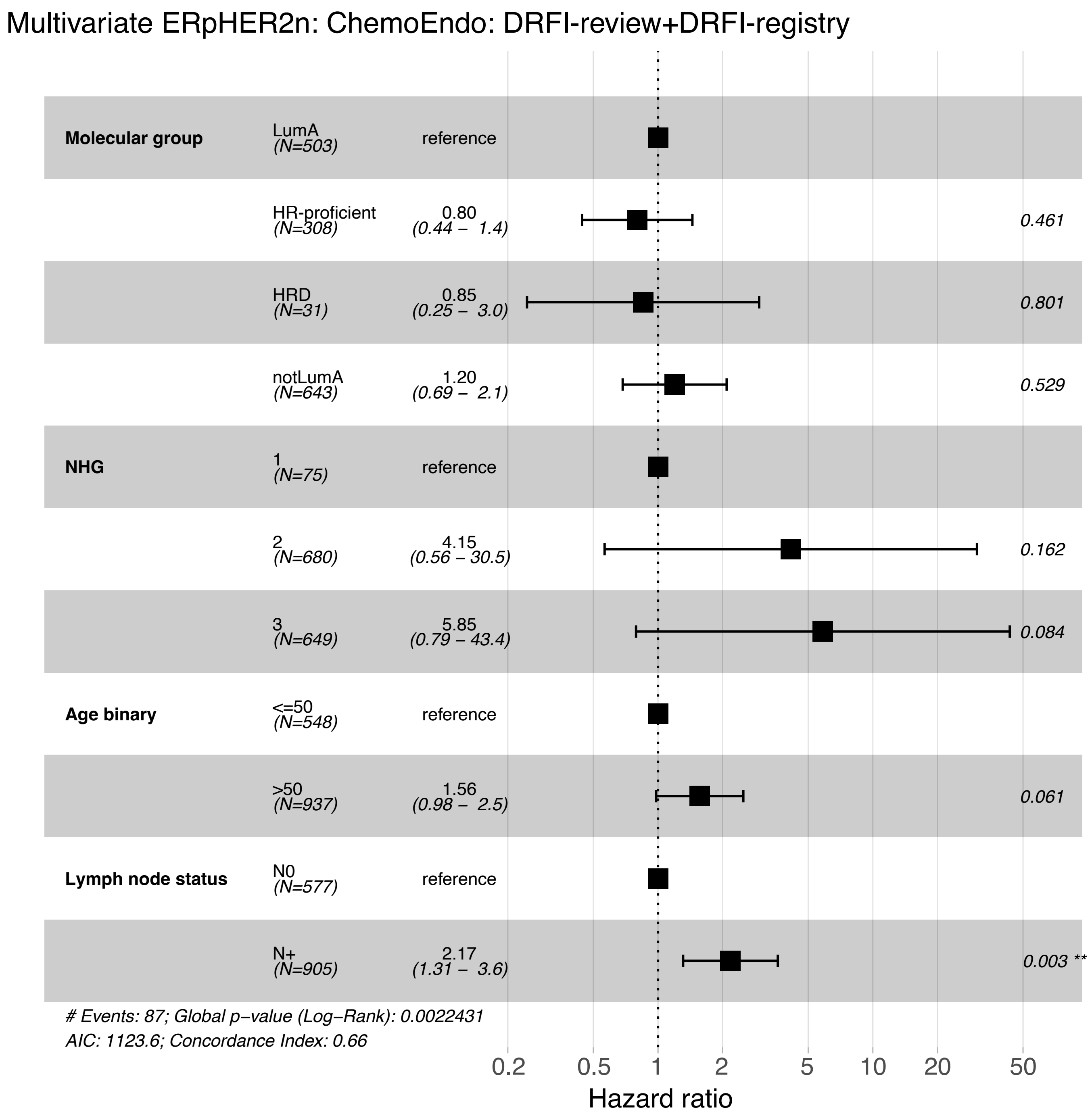

Multivariate ERpHER2n: ChemoEndo: DRFI-review+DRFI-registry
Molecular group
LumA (N=503) reference
HR-proficient (N=308) 0.80 (0.44 – 1.4) 0.461
HRD (N=31) 0.85 (0.25 – 3.0) 0.801
notLumA (N=643) 1.20 (0.69 – 2.1) 0.529
NHG
1 (N=75) reference
2 (N=680) 4.15 (0.56 – 30.5) 0.162
3 (N=649) 5.85 (0.79 – 43.4) 0.084
Age binary
<=50 (N=548) reference
>50 (N=937) 1.56 (0.98 – 2.5) 0.061
Lymph node status
N0 (N=577) reference
N+ (N=905) 2.17 (1.31 – 3.6) 0.003 **
Events: 87; Global p−value (Log−Rank): 0.0022431
AIC: 1123.6; Concordance Index: 0.66
0.2 0.5 1 2 5 10 20 50
Hazard ratio
