# Supplementary material for: Homologous recombination deficiency in primary ER-positive and HER2-negative breast cancer
Source: Commun Med (Lond). 2026 Feb 16;6:118. doi: 10.1038/s43856-026-01385-0 (PMC12909304; doi:10.1038/s43856-026-01385-0)
Supplement: Supplementary file 2 — Description of Additional Supplementary files [file 43856_2026_1385_MOESM2_ESM.pdf]

Description of Additional Supplementary Files

Supplementary Data 1. Source Data.

Supplementary Data 2. Excel file including supplementary patient data and SBS catalogue.

Supplementary Data 3. Excel file with tables describing differentially expressed genes and GSEA pathway results between HRD and HR-proficient tumors in specific sample subsets.

Supplementary Data 4. Excel file listing analyzed DNA repair and HRD gene sets.

Supplementary Data 5. Excel file listing detected HRD inactivation mechanisms.
